# Antiproliferative Aspidosperma-Type Monoterpenoid Indole Alkaloids from *Bousigonia mekongensis* Inhibit Tubulin Polymerization

**DOI:** 10.3390/molecules24071256

**Published:** 2019-03-31

**Authors:** Yu Zhang, Masuo Goto, Akifumi Oda, Pei-Ling Hsu, Ling-Li Guo, Yan-Hui Fu, Susan L. Morris-Natschke, Ernest Hamel, Kuo-Hsiung Lee, Xiao-Jiang Hao

**Affiliations:** 1 Key Laboratory of Phytochemistry and Plant Resources in West China, Kunming Institute of Botany, Chinese Academy of Sciences, Kunming 650201, China; zhangyu@mail.kib.ac.cn (Y.Z.); guolingli@mail.kib.ac.cn (L.-L.G.); fuyanhui80@163.com (Y.-H.F.); 2 Natural Product Research Laboratories, UNC Eshelman School of Pharmacy, University of North Carolina, Chapel Hill, NC 27599, USA; peiling96@livemail.tw (P.-L.H.); susan_natschke@unc.edu (S.L.M.-N.); 3 Graduate School of Pharmacy, Meijo University, 150 Yagotoyama, Tempaku-ku, Nagoya, Aichi 468-8503, Japan; oda@meijo-u.ac.jp; 4 Screening Technologies Branch, Developmental Therapeutics Program, Division of Cancer Treatment and Diagnosis, Frederick National Laboratory for Cancer Research, National Cancer Institute, Frederick, MD 21702, USA; hamele@dc37a.nci.nih.gov; 5 Chinese Medicine Research and Development Center, China Medical University and Hospital, 2 Yuh-Der Road, Taichung 40447, Taiwan

**Keywords:** aspidosperma-type, monoterpenoid indole alkaloids, antiproliferative activity, tubulin inhibitor, *Bousigonia mekongensis*

## Abstract

Monoterpenoid indole alkaloids are structurally diverse natural products found in plants of the family Apocynaceae. Among them, vincristine and its derivatives are well known for their anticancer activity. *Bousigonia mekongensis*, a species in this family, contains various monoterpenoid indole alkaloids. In the current study, fourteen known aspidosperma-type monoterpenoid indole alkaloids (**1**–**14**) were isolated and identified from a methanol extract of the twigs and leaves of *B. mekongensis* for the first time. Among them, compounds **3**, **6**, **9**, and **13** exhibited similar antiproliferative activity spectra against A549, KB, and multidrug-resistant (MDR) KB subline KB-VIN cells with IC_50_ values ranging from 0.5–0.9 μM. The above alkaloids efficiently induced cell cycle arrest at the G2/M phase by inhibiting tubulin polymerization as well as mitotic bipolar spindle formation. Computer modeling studies indicated that compound **7** likely forms a hydrogen bond (H-bond) with α- or β-tubulin at the colchicine site. Evaluation of the antiproliferative effects and SAR analysis suggested that a 14,15-double bond or 3α-acetonyl group is critical for enhanced antiproliferative activity. Mechanism of action studies demonstrated for the first time that compounds **3**, **4**, **6**, **7**, and **13** efficiently induce cell cycle arrest at G2/M by inhibiting tubulin polymerization by binding to the colchicine site.

## 1. Introduction

Microtubule-binding agents have been developed as an effective therapy in cancer treatment due to the key roles of microtubules in cell proliferation, signal transduction, and cell migration [1]. Currently, many microtubule-binding agents, including taxanes, vinca alkaloids, epothilones, halichondrins, maytansinoids, colchicine-site binding agents, and others, have been discovered from natural products and later progressed to clinical studies and clinical use [2]. However, innate and acquired drug resistance, especially multidrug resistance (MDR), are major obstacles in cancer chemotherapy [3]. Overexpression of P-glycoprotein (P-gp) encoded by the ABCB1 gene leads to poor disease prognosis, and most clinical antimicrotubule drugs, including paclitaxel (PXL), vincristine (VIN), halichondrin B, and their analogs, are P-gp substrates [4]. A recent study indicated that antimitotic agents that target the colchicine site (CS) on the α/β-tubulin dimer were generally active in cells overexpressing βIII-tubulin, which is important in tumor aggressiveness and resistance to chemotherapy [5]. Hence, the discovery of other antimitotic CS-targeting agents might be a valuable approach for effective cancer chemotherapy, especially agents with enhanced tumor specificity and insensitivity to chemoresistance mechanisms.

Monoterpenoid indole alkaloids (MIAs) are secondary metabolites characteristic of plants in the family Apocynaceae [6]. Among them, the vinca alkaloids exhibit significant anticancer activity and vincristine, vinblastine, vinorelbine, vindesine, and vinflunine have been approved for clinical use in the treatment of hematological and lymphatic neoplasms [7]. Previous chemical studies on MIAs mostly focused on the dimeric compounds (vindoline-catharanthine), which generally exhibit superior anticancer activities compared with the corresponding monomeric units. Aspidosperma-type MIAs contain only the vindoline structural unit found in vincristine and are widely distributed in the genera *Tabernaemontana*, *Melodinus*, and *Bousigonia* (family Apocynaceae) [6]. In prior biological activity studies, several aspidosperma-type MIAs, such as jerantinines A, B, and E from *T. corymbose*, displayed significant antiproliferative activity against cancer cells [8]. Further mechanistic studies demonstrated that these alkaloids significantly arrested cells at the G2/M phase by inhibiting tubulin polymerization and, thus, they merit development as potential chemotherapeutic agents [9,10,11].

**Figure 1:**
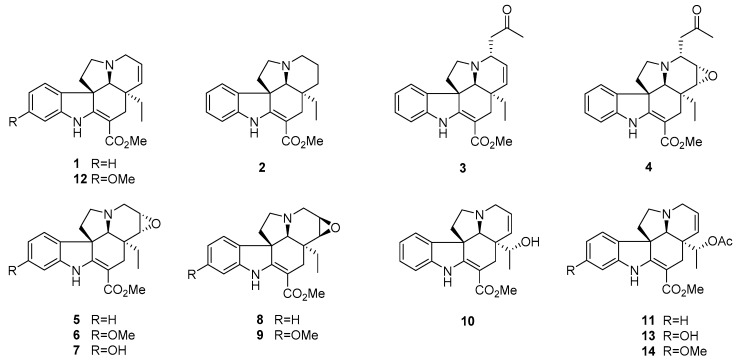
Structures of aspidosperma-type MIAs (**1**–**14**) from *B. mekongensis*.

The genus *Bousigonia* (family Apocynaceae) contains only two species (*B. mekongensis* and *B. angustifolia*), distributed mainly in Southern China, Laos, and Vietnam [12]. Previous chemical investigation on this genus conducted in our group resulted in a series of new eburnamine-aspidospermine-type bisindole alkaloids and aspidosperma-type MIAs [13,14,15]. As part of our ongoing work, the present study investigated the antiproliferative activity of aspidosperma-type MIAs (**1**–**14**) against five cancer cell lines, A549, MDA-MB-231, KB, P-gp-overexpressing KB subline KB-VIN, and MCF-7, as well as a primary structure activity relationship (SAR) analysis including computer modeling. The detailed mechanisms of action of these alkaloids (Figure 1) were also further investigated and are described herein.

## 2. Results and Discussion

### 2.1. Chemistry

Fourteen aspidosperma-type monoterpenoid indole alkaloids, tabersonine (**1**) [16], vincadifformine (**2**) [16], 3*α*-acetonyl-tabersonine (**3**) [17], melodinine S (**4**) [18], lochnericine (**5**) [19], 14,15-*α*-epoxy-11-methoxytabersonine (**6**) [20], 11-hydroxylochnericine (**7**) [20], pachysiphine (**8**) [21], lochnerinine (**9**) [22], 19-(*R*)-hydroxytabersonine (**10**) [23], 19-(*R*)-acetoxytabersonine (**11**) [24], 11-methoxytabersonine (**12**) [16], 19-(*R*)-acetoxy-11-hydroxytabersonine (**13**) [24], 19-(*R*)-acetoxy-11-methoxytabersonine (**14**) [24] were isolated and identified from the methanol extract of *B. mekongensis* for the first time by a combination of chromatographic and spectroscopic methods (Figure 1, Tables S1–S3). The effects of the substituents at positions C-3, C-11, C-14/C-15, and C-19 were evaluated by considering the hydrophilic or hydrophobic properties.

### 2.2. Antiproliferative Activity of Compounds 1–14

We tested the fourteen known aspidosperma-type alkaloids (**1**–**14**) against chemosensitive KB (originally isolated from epidermoid carcinoma of the nasopharynx) and P-gp over-expressing multidrug-resistant (MDR) KB subline KB-VIN, as well as three additional human cancer cell lines, A549 (lung carcinoma), MDA-MB-231 (triple negative breast cancer), and MCF-7 (estrogen receptor-positive breast cancer), using a sulforhodamine B (SRB) assay (Table 1). Vincristine (VIN), paclitaxel (PXL), and a CS agent combretastatin A-4 (CA-4) were also tested as positive controls.

### 2.3. Biological Activity Comparison

While six alkaloids (**2**, **5**, **8**, **10**, **11**, and **14**) were essentially inactive, the remaining eight alkaloids (**1**, **3**, **4**, **6**, **7**, **9**, **12**, and **13**) exhibited antiproliferative activity against all cell lines tested in this study, including the MDR subline KB-VIN (Table 1). Particularly, alkaloids **3**, **6**, **9**, and **13** showed substantial potency against KB-VIN cells with IC_50_ values ranging from 0.5–0.7 μM. Notably, all eight active alkaloids were effective against both chemosensitive and MDR cells, as compared with well-known P-gp substrates VIN and PXL, which required 600-fold higher concentration against KB-VIN cells. The results indicate that the tested alkaloids are not substrates of P-gp and, thus, could be effective against tumors expressing the MDR phenotype.

Alkaloids **1**–**14** have the same carbon skeleton but differ in the substituents or oxidation state at various positions. Based on the antiproliferative activity data, a 14,15-double bond is critical for activity as alkaloids **2**, **5**, and **8** with a 14,15-single bond lost potency. Addition of a 3α-acetonyl group led to greatly increased potency (compare **3** versus **1**, **4** versus **5**). Potency was lost when the α-carbon of the ethyl group attached at C-19 of **1** was substituted with a hydroxyl (**10**) or acetoxy (**11**) moiety. The presence of a hydroxyl group at C-11 had a significant effect on potency (compare **7** versus **5**, **13** versus **11**), while a methoxy group at the same position led to both increased (~60-fold rise between **5** and **6**, **8** and **9**) and negligible (compare **1** versus **12**, **14** and **11**) potency. 

Taken together, a 14,15-double bond or 3α-acetonyl group was required for antiproliferative activity against human cancer cell lines, including the MDR subline KB-VIN. A hydroxyl group at C-11 is necessary, while the effect of a methoxy group at C-11 was dependent on the parent skeleton. The compatibility of the synergistic groups will be considered in subsequent SAR studies. 

### 2.4. Mechanisms of Action of 3, 4, 6, 7, and 13 in KB-VIN Cells

Bioactive analogs **6** and **7** were tested for inhibitory effects on tubulin assembly as well as inhibition of [^3^H] colchicine binding to tubulin in a cell-free system, using highly purified bovine brain tubulin (Table 1). The results showed that alkaloid **6** strongly inhibited tubulin assembly with an EC_50_ (50% effective concentration for inhibiting tubulin assembly) value of 0.7 μM and inhibited the binding of colchicine to tubulin by 54%, while **7** showed moderate inhibition of tubulin assembly with an EC_50_ value of 4.6 μM. The data for **6** and **7** indicate that both alkaloids bind to tubulin and inhibit its assembly, and these effects are closely related to their antiproliferative activities against tumor cells. Thus, we further investigated whether the inhibition of tubulin polymerization was the major action of these alkaloids.

**Figure 2:**
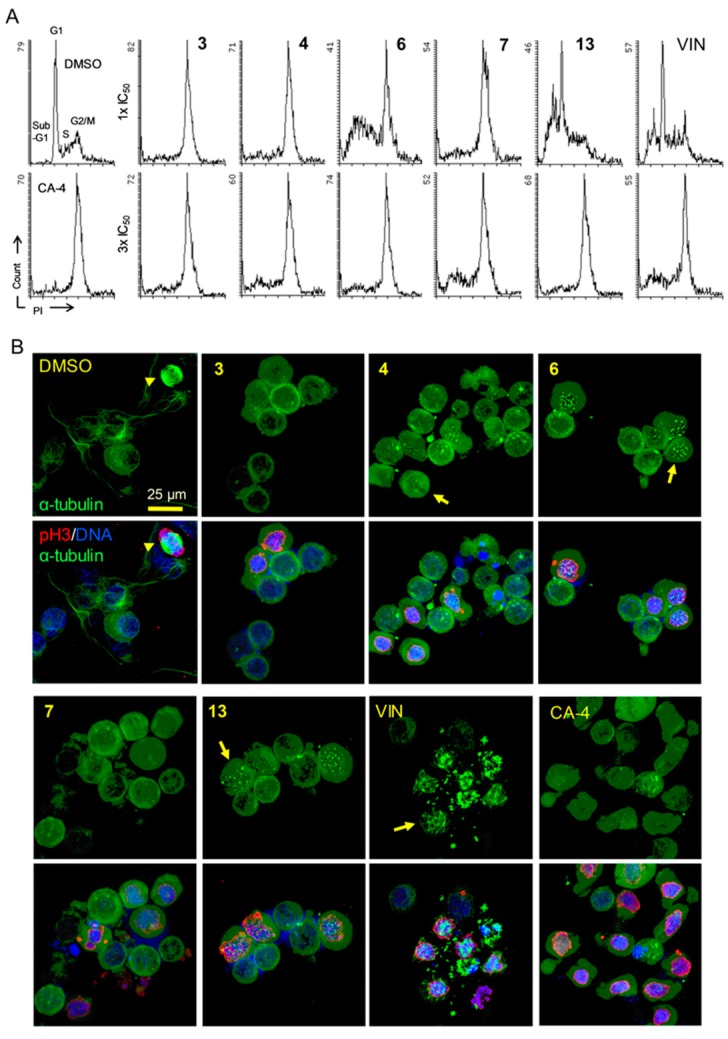
Mitotic defects in KB-VIN cells treated by compounds. (**A**) Vincristine-resistant subline KB-VIN cells were treated with compounds for 24 h at a concentration of one- or three-fold IC_50_ (1× IC_50_ or 3 × IC_50_). CA-4 at 0.2 µM was used as a colchicine-type tubulin polymerization inhibitor. Cell cycle distributions (sub-G1, G1, S, G2/M) were analyzed using flow cytometry after staining cells with propidium iodide (PI). (**B**) KB-VIN cells were treated with compounds for 24 h at a concentration of 3 × IC_50_. CA-4 was used at 0.2 μM. Fixed cells were stained with antibodies to α-tubulin (green) and phospho-histone H3 (pH3, red), and DAPI was used for DNA (blue). Stained cells were observed by confocal fluorescence microscope. The represented image is a projection of 15~20 optical sections acquired at 0.5~1 µm intervals. Normal mitotic spindle formation (arrow head) in control (DMSO) and dotted tubulin aggregations without spindles (**4**, **6**, **13**) or with multipolar spindles (VIN) were observed (arrows). Bar, 0.025 mm. Additional images are available in Supplementary Figure S1.

CA-4, a colchicine site (CS) agent, and other tubulin polymerization inhibitors induce cell cycle arrest at G2/M. Accordingly, we investigated whether the MIAs affected the cell cycle progression. KB-VIN cells were treated with compounds **3**, **4**, **6**, **7**, and **13** at their IC_50_ (1 × IC_50_) or three-fold IC_50_ (3 × IC_50_) concentration, and the cell cycle progression was analyzed by flow cytometer (Figure 2A). Expectedly, the accumulation of cells in the G2/M phase was observed in cells treated with alkaloids **3**, **4**, **6**, **7**, and **13**, as compared with CA-4 and vincristine (VIN). 

To determine whether the cell cycle arrest was due to an antimicrotubule effect, cells treated with compounds were analyzed by immunocytochemistry using antibodies to α-tubulin for microtubules and mitotic spindles, Ser10-phosphorylated histone H3 (pH3) for the condensed chromatins and 4′,6-diamidino-2-phenylindole (DAPI) for DNA. In cells treated with alkaloids **3**, **4**, **6**, **7**, and **13**, dotted tubulin aggregations without spindles were seen in the pH3-positive mitotic cells, while microtubules were undetectable in pH3-negative interphase cells (Figure 2B). These observations demonstrated that the tested alkaloids inhibited tubulin polymerization in both interphase and mitosis for bipolar spindle formation, inducing cell cycle arrest at G2/M, probably at prometaphase. In addition, alkaloid **7** was less active than **6**, which corresponded to the inhibitory activity observed in cell-based proliferation and cell-free tubulin assembly assays. Thus, we concluded that these alkaloids are tubulin polymerization inhibitors. Furthermore, the immunocytochemical data suggest that alkaloids **3, 4**, **6**, **7**, and **13** probably interact with tubulin in a biological manner similar to that of CA-4. These results agreed with those in a recent study on aspidosperma-type MIAs, such as jerantinine A, which potentially inhibit tubulin polymerization by binding to the CS on tubulin dimers [25].

**Figure 3:**
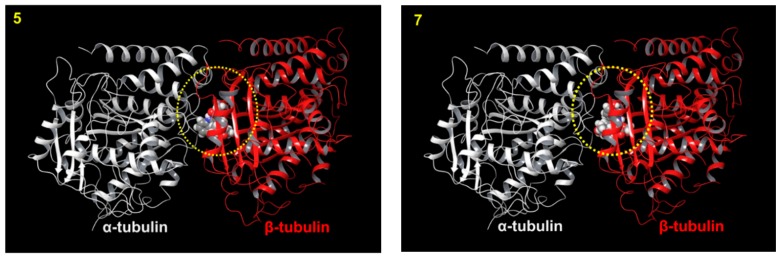
Predicted docking models for **5** and **7** binding to tubulin. Top 1 ranked docking models of **5** and **7** (sphere in 3D with gray in carbon, proton in white, oxygen in red, nitrogen in blue) in the colchicine site (CS, yellow circle) of the tubulin crystal structure (α and β tubulin heterodimer: α- (white) and β-tubulin (red)) (PDB: 1SA0) are shown as a ribbon diagram.

**Figure 4:**
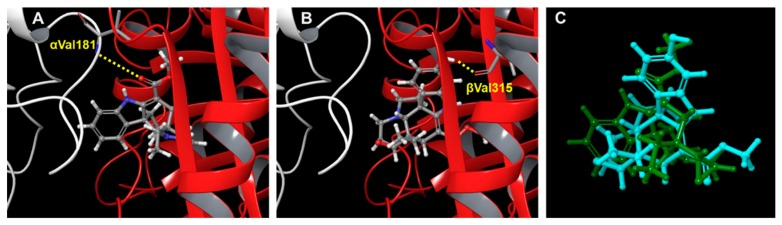
Predicted docking models for **5** and **7** binding in the CS. The crystal structures (PDB: 1SA0) of α- (white) and β-tubulin (red) are shown as ribbon diagrams. The distances calculated to be less than 5 Å between heavy atoms are represented by dashed lines. Docking models of compounds (gray skeleton with oxygen in red and nitrogen in blue) **5** (**A**) and **7** (**B**) in the CS are shown. Superimposition of docked compound **5** or **7** shows H-bonds with the side chain of αVal181 or βVal315, respectively. (**C**) Comparison of docking mode of **5** (green) with that of **7** (blue) in CS.

Accordingly, we performed molecular docking studies to predict how alkaloid **7** binds to the tubulin dimer. An inactive alkaloid **5** was used as a comparison compound. In the tubulin binding assay described above (Table 1), CA-4 totally inhibited colchicine binding to tubulin, while **5** or **7** inhibited binding with 0 or 35% ICB value, respectively. Thus, compared with CA-4, alkaloid **5** and its hydroxyl analog **7** might bind differently to the CS. As expected, an overview of the predicted binding modes of **5** and **7** in the crystal structure of the α/β-tubulin dimer revealed considerable differences (Figure 3 and Figure 4). The docked model of **5** showed an H-bond between the carbonyl oxygen and the side chain of Val181 on α-tubulin (αVal181), while that of **7** showed an H-bond between the C-11 hydroxyl group and the side chain of Val315 on β-tubulin (βVal315). Interestingly, in a cell-free tubulin assembly assay, compound **7** (EC_50_ 4.6 µM) was more potent than **5** (EC_50_ > 40 µM). These analyses suggested that the binding mode of **5** was insufficient to inhibit tubulin assembly. The docking model also predicted that active compound **13** (IC_50_ 0.6~6.7 µM) forms an H-bond with βAsn249, while less active compounds **4** (IC_50_ 5.6~10.0 µM) and **11** (IC_50_ > 26.5 µM) form H-bonds with αThr179 and αSer178, respectively (Supplementary Figure S2). However, H-bonding with αVal181 may also be important and depends on a steric hindrance due to the parent skeleton (compare **3** versus **5**). This docking model suggested that the force of the H-bond between βVal315 or βAsn249 and the C-11 hydroxyl group of **7** might be critical for greater inhibition of tubulin assembly, which is also reflected in greater antiproliferative activity.

## 3. Materials and Methods

### 3.1. General Experimental Procedures

All chemicals and solvents were used as purchased. ESIMS data were obtained on a Finnigan MAT 90 spectrometer. NMR spectra were recorded on Bruker DRX-500 and Avance III -600 NMR spectrometers using TMS as an internal standard. All chemical shifts are reported in ppm, and apparent scalar coupling constants *J* are given in Hertz. Silica gel (300–400 mesh, Qingdao Marine Chemical Inc., Shandong, China), silica gel H (10–40 μm, Qingdao Marine Chemical Inc.), Lichroprep RP-18 gel (40–63 μm, Merck, Darmstadt, Germany), and Sephadex LH-20 (40–70 μm, Amersham Biosciences, Waltham, MA, USA) were used for column chromatography (CC). All target compounds were characterized and determined to be at least >95% pure by ^1^H NMR and analytical HPLC.

### 3.2. Plant Material

The twigs and leaves of *B. mekongensis* were collected during April 2010 from Mengla County, Yunnan Province, PR China and identified by Mr. Jing-Yun Cui, Xishuangbanna Tropical Plant Garden. A voucher specimen (No. CUI20100419) has been deposited at the State Key Laboratory of Phytochemistry and Plant Resources in West China, Kunming Institute of Botany, Chinese Academy of Science (CAS).

### 3.3. Extraction and Isolation

The dried twigs and leaves of *B. mekongensis* (12 kg) were extracted with CH_3_OH, the crude extract was dissolved in aqueous solution, and then the pH was adjusted to 2 by adding saturated tartaric acid with adequate stirring. The acidic mixture was defatted with petroleum ether (PE) and then extracted with CHCl_3_. The aqueous phase was basified to pH~10 with saturated Na_2_CO_3_ and then extracted with CHCl_3_ to obtain crude alkaloids. The crude alkaloids (60 g) were separated on a silica gel column (200–300 mesh; CHCl_3_/CH_3_OH, 1:0 → 0:1), yielding five major fractions (Fr 1–5). Fraction 1 (12.8 g) was chromatographed with a series of silica gel columns (CHCl_3_/acetone and CHCl_3_/CH_3_OH) to afford compounds **3** (21 mg) and **4** (16 mg). Fraction 2 (11.2 g) was further chromatographed on a reversed-phase C_18_ silica gel medium-pressure column (CH_3_OH/H_2_O, 1:1 → 1:0) to give four fractions (Fr 2A–2D). Fraction 2C (3.2 g) was separated on a silica gel column (300–400 mesh; PE/ acetone, 3:1), yielding three fractions (Fr 2C1–2C3). Compound **1** (16 mg) was separated from fraction 2C2 (760 mg) by semipreparative HPLC using a Waters XBridge C_18_ (10 × 250 mm, 5 µm) column with 70% CH_3_CN/H_2_O with added 0.1 *v*/*v* diethylamine. Compound **11** (36 mg) was obtained from fraction 2C3 (368 mg) by semipreparative HPLC using a Waters XBridge C_18_ (10 × 250 mm, 5 µm) column with 70% CH_3_OH/H_2_O with added 0.1 *v*/*v* diethylamine. Fraction 3 (9.8 g) was further chromatographed over a reversed-phase C_18_ silica gel medium-pressure column (CH_3_OH/H_2_O, 1:1 → 1:0) to give four fractions (Fr 3A–3D). Fraction 3A (480 mg) was separated by semipreparative HPLC using a Waters XBridge C_18_ (10 × 250 mm, 5 μm) column with 45% CH_3_OH/H_2_O to give compounds **5** (21 mg), **10** (4.0 mg) and **12** (28 mg). Fraction 3C (780 mg) was purified using a Sephadex LH-20 column eluted with CH_3_OH, followed by semipreparative HPLC using a Waters XBridge C_18_ (19 × 250 mm, 5 μm) column with 70% CH_3_OH/H_2_O to afford compounds **8** (22 mg) and **14** (48 mg). Fraction 4 (9.8 g) was further chromatographed over a reversed-phase C_18_ silica gel medium-pressure column (CH_3_OH/H_2_O, 1:1 → 1:0) to give four fractions (Fr 4A–4D). Fr 4A (518 mg) was separated by semipreparative HPLC using a Waters XBridge C_18_ (10 × 250 mm, 5 μm) column with 40% CH_3_OH/H_2_O to afford compounds **2** (9 mg), **6** (28 mg), **7** (20 mg), and **9** (12 mg). Fr 4B (300 mg) was further purified using a Sephadex LH-20 column eluted with CH_3_OH to afford **13** (35 mg).

### 3.4. Antiproliferative Activity Assay

Antiproliferative activity was determined by the sulforhodamine B (SRB) colorimetric assay as previously described [26]. In brief, human tumor cell lines were cultured in RPMI-1640 medium containing 2 mM l-glutamine and 25 mM HEPES (Gibco), supplemented with 10% fetal bovine serum (Speciality Media), 100 µg/mL streptomycin, 100 IU/mL penicillin, and 0.25 µg/mL amphotericin B (Corning). MDR stock cells (KB-VIN) were maintained in the presence of 100 nM vincristine (VIN) (Sigma-Aldrich, Saint Louis, MI, USA).

Freshly trypsinized cell suspensions were seeded in 96-well microtiter plates at densities of 4000–11,000 cells per well (based on the cell lines) with compounds. After 72 h in culture with test compounds, cells were fixed in 10% trichloroacetic acid followed by staining with 0.04% sulforhodamine B (Sigma-Aldrich). The bound SRB was solubilized in 10 mM Tris-base and the absorbance at 515 nm was measured using a Microplate Reader (ELx800, Bio-Tek Instruments, Winooski, VT, USA) operated by Gen5 software (BioTek) after solubilizing the protein-bound dye with 10 mM Tris base. The mean IC_50_ is the concentration of agent that reduced cell growth by 50% compared with vehicle (DMSO) control under the experimental conditions and is the average from at least three independent experiments with duplicate samples.

The following human tumor cell lines were used in the assay: A549 (lung carcinoma), MDA-MB-231 (triplen-egative breast cancer), KB (originally isolated from epidermoid carcinoma of the nasopharynx), KB-VIN (VIN-resistant KB subline showing MDR phenotype by overexpressing P-gp), MCF-7 (estrogen receptor (ER)-positive, HER2-negative breast cancer). It should be noted that we confirmed the KB and KB-VIN cell lines were identical to AV-3 (ATCC number, CCL-21) as a HeLa (cervical carcinoma) contaminant by short tandem repeat (STR) profiling. All cell lines were obtained from the Lineberger Comprehensive Cancer Center (UNC-CH) or from ATCC (Manassas, VA), except KB-VIN, which was a generous gift from Professor Y.-C. Cheng (Yale University). Paclitaxel was purchased from Sigma-Aldrich.

### 3.5. Tubulin Assays

Inhibitory effects of compounds on tubulin assembly were evaluated using electrophoretically homogeneous bovine brain tubulin as described previously [27,28] using 1.0 mg/mL (10 μM) tubulin. The turbidity development as tubulin assembly was initiated by adding 0.4 mM GTP and followed for 20 min at 30 °C following a rapid temperature jump. Compound concentrations as EC_50_ values that inhibited the increase in turbidity by 50% relative to a control sample were determined. In colchicine inhibition assays, tubulin (1.0 μM) was incubated with 5.0 μM [^3^H] colchicine and 5.0 μM test compound at 37 °C for 10 min, when about 40–60% of maximum colchicine binding occurs in control samples.

### 3.6. Cell Cycle Analysis

KB-VIN cells were seeded in 12-well plates at a density of 1 × 10^5^ per well and incubated overnight. After 24 h of treatment with tested compounds at a concentration of one- or three-fold IC_50_, the cells were harvested and fixed in 70% EtOH at −20 °C overnight followed by staining with propidium iodide (PI) containing RNase (BD Pharmingen, Franklin Lakes, NJ, USA) for 30 min at 37 °C. The DNA contents of stained cells were analyzed by flow cytometer (LSRFortessa, BD Biosciences) controlled by FACSDiva software (BD Biosciences). Paclitaxel (PXL) was used at 3 or 6 µM. Combretastatin A-4 (CA-4) was purchased from Sigma-Aldrich and used at 0.2 μM. Experiments were repeated a minimum of two times.

### 3.7. Immunofluorescence Staining

Immunocytochemical analysis was performed as previously described [26]. KB-VIN cells were grown on an 8-well chamber slide (Lab-Tech) for 24 h prior to treatment with the compound at a concentration of three-fold IC_50_. CA-4 was used at 0.2 μM (Figure 2A and Supporting Information, Supplementary Figure S1). After treatment of cells with the agent for 24 h, cells were fixed with 4% paraformaldehyde in PBS and permeabilized with 0.5% Triton X-100 in PBS. Fixed cells were labeled with mouse monoclonal antibody to α-tubulin (B5-1-2, Sigma) and rabbit IgG to Ser10-phosphorylated histone H3 (p-H3) (#06570, EMD Millipore), followed by FITC-conjugated antibody to mouse IgG (Sigma) and Alexa Fluor 549-conjugated antibody to rabbit IgG (Life Technologies). Nuclei were labeled with DAPI (Sigma). Fluorescently-labeled cells were observed using a confocal microscope (Zeiss, LSM700) with ZEN (black edition) software (Zeiss). The 15~20 optical sections acquired at 0.5~1 µm intervals were stacked and reconstructed using ZEN (black edition) software. Experiments were repeated at least twice for each compound. Final images were prepared using Adobe Photoshop CS3.

### 3.8. Computer Modeling

GOLD 5.1 software with default settings was used to predict the three-dimensional (3D) structures of tubulin-ligand complexes [29]. The human tubulin 3D structure (TUBA1A and TUBB2B) used in this study was built from a Protein Data Bank entry (PDB ID: 1SA0) [30]. Absent hydrogen atoms in the crystal structure were added computationally by Hermes software 5.1 version. The active site radius was set to 10.0 Å, and the active site center was defined as the ligand center in 1SA0. The docking calculations used the quantum chemically-optimized ligand structures as the initial structures. Structural optimizations of ligands were performed with B3LYP/6-311+G (df, p) using Gaussian 09, Revision B.01 [31].

## 4. Conclusions

Fourteen aspidosperma-type monoterpenoid indole alkaloids (**1**–**14**) were isolated from a methanol extract of the twigs and leaves of *Bousigonia mekongensis*. All isolates were evaluated for antiproliferative activity against five human tumor cell lines, including the MDR subline KB-VIN. Alkaloids **3**, **4**, **6**, **7**, and **13** showed significant antiproliferative effects against all five tumor cell lines. Because activity was retained against KB-VIN, the active alkaloids are not P-gp substrates and, thus, could be effective against tumors expressing the MDR phenotype. SAR studies suggested that the presence of a 14,15-double bond or 3α-acetonyl group is critical for antiproliferative activity. Furthermore, mechanistic studies revealed for the first time that the five active alkaloids cause significant arrest of the cell cycle progression at the G2/M cell cycle phase via inhibition of tubulin polymerization. In addition, the compounds interact with tubulin in a manner distinct from that of CA-4.

## Figures and Tables

**Table 1:** Antiproliferative activity and effect on tubulin assembly.

Compound	IC_50_ (μM) *^a^*					Tubulin Assay	
	A549	MDA-MB-231	KB	KB-VIN	MCF-7	ITA*^d^* (μM)	ICB*^e^* (%)
**1**	6.9 ± 0.1	5.9 ± 0.0	5.5 ± 0.0	7.4 ± 0.3	7.2 ± 0.4	NT*^b^*	NT
**2**	>40	>40	>40	>40	>40	NA*^c^*	NA
**3**	0.6 ± 0.0	0.9 ± 0.0	0.6 ± 0.0	0.6 ± 0.1	2.2 ± 0.2	NT	NT
**4**	5.6 ± 0.0	8.2 ± 0.8	5.8 ± 0.3	6.3 ± 0.2	10.0 ± 0.9	NT	NT
**5**	>40	>40	>40	>40	>40	NA	NA
**6**	0.5 ± 0.0	0.8 ± 0.0	0.5 ± 0.0	0.5 ± 0.0	0.8 ± 0.0	0.7 ± 0.0	54 ± 0.8
**7**	5.6 ± 0.1	9.8 ± 0.9	5.4 ± 0.3	6.1 ± 0.4	10.8 ± 0.8	4.6 ± 0.1	35 ± 1
**8**	27.7 ± 0.1	34.5 ± 0.2	>40	>40	>40	NA	NA
**9**	0.8 ± 0.0	6.0 ± 0.8	0.9 ± 0.0	0.7 ± 0.0	10.2 ± 0.5	NT	NT
**10**	>40	>40	>40	>40	>40	NA	NA
**11**	>40	>40	26.5 ± 2.5	>40	>40	NA	NA
**12**	5.2 ± 0.1	6.8 ± 1.1	5.4 ± 0.4	5.8 ± 0.1	8.2 ± 0.9	NT	NT
**13**	0.7 ± 0.1	0.9 ± 0.0	0.7 ± 0.0	0.7 ± 0.0	6.7 ± 0.1	NT	NT
**14**	>40	>40	>40	>40	>40	NA	NA
VIN (nM)	22.9 ± 2.4	32.0 ± 0.5	4.4 ± 0.1	2479.2 ± 28.2	7.3 ± 0.2	NT	NT
PXL (nM)	4.5 ± 0.9	7.0 ± 0.9	3.7 ± 1.1	2357.7 ± 59.5	8.8 ± 1.0	NT	NT
CA-4 (nM)	5.5 ± 0.1	8.2 ± 0.5	3.6 ± 0.1	3.8 ± 0.1	487.4 ± 11	0.7 ± 0.0	100 ± 0.4

^a^ Antiproliferative activity as IC_50_ values for each cell line, the concentration of compound that caused a 50% reduction relative to untreated cells determined by the SRB assay. ^b^ NT, not tested. ^c^ NA, not active (IC_50_ > 40 µM). ^d^ Inhibition of purified tubulin assembly, EC_50_ (µM) values of 50% inhibition (ITA). ^e^ Percent inhibition of 5 μM [^3^H] colchicine binding to 1 μM tubulin in the presence of 5 μM test compound (ICB).

## Supplementary Materials

The following are available online at https://www.mdpi.com/1420-3049/24/7/1256/s1, Figure S1: Inhibition of tubulin polymerization by compounds, Figure S2: Predicted docking models for **3**, **4**, **11**, and **13** binding in the CS; Tables S1–S3: ^1^H and ^13^C NMR data (*δ*) for compounds **1**–**14**.
