# Supplementary material for: Antiproliferative Aspidosperma-Type Monoterpenoid Indole Alkaloids from Bousigonia mekongensis Inhibit Tubulin Polymerization
Source: Molecules. 2019 Mar 31;24(7):1256. doi: 10.3390/molecules24071256 (PMC6480704; doi:10.3390/molecules24071256)
Supplement: Supplementary file 1 [file molecules-24-01256-s001.pdf]

Supporting Information

for

Cytotoxic aspidosperma-type monoterpenoid indole alkaloids from *Bousigonia mekongensis* inhibit tubulin polymerization

Yu Zhang[a,b], Masuo Goto[b,**], Akifumi Oda[c], Pei-Ling Hsu[b], Ling-Li Guo[a], Yan-Hui Fu[a], Susan L. Morris-Natschke[b], Ernest Hamel[d], Kuo-Hsiung Lee[b,e,**] Xiao-Jiang Hao[a,*]

[a]*State Key Laboratory of Phytochemistry and Plant Resources in West China, Kunming Institute of Botany, Chinese Academy of Sciences, Kunming 650201, Yunnan, People's Republic of China*

[b]*Natural Product Research Laboratories, UNC Eshelman School of Pharmacy, University of North Carolina, Chapel Hill, NC 27599, USA*

[c]*Graduate School of Pharmacy, Meijo University, 150 Yagotoyama, Tempaku-ku, Nagoya, Aichi 468-8503, Japan*

[d]*Screening Technologies Branch, Developmental Therapeutics Program, Division of Cancer Treatment and Diagnosis, Frederick National Laboratory for Cancer Research, National Cancer Institute, Frederick, Maryland, 21702, USA*

[e]*Chinese Medicine Research and Development Center, China Medical University and Hospital, 2 Yuh-Der Road, Taichung, 40447, Taiwan*

Contents:

Supplementary Figure S1. **Inhibition of tubulin polymerization by compounds.**

Supplementary Figure S2. **Predicted docking models for 3, 4, 11, and 13 binding in the CS.**

Supplementary Tables S1-S3. **^{1}H and ^{13}C NMR data (δ) for compounds 1-14.**

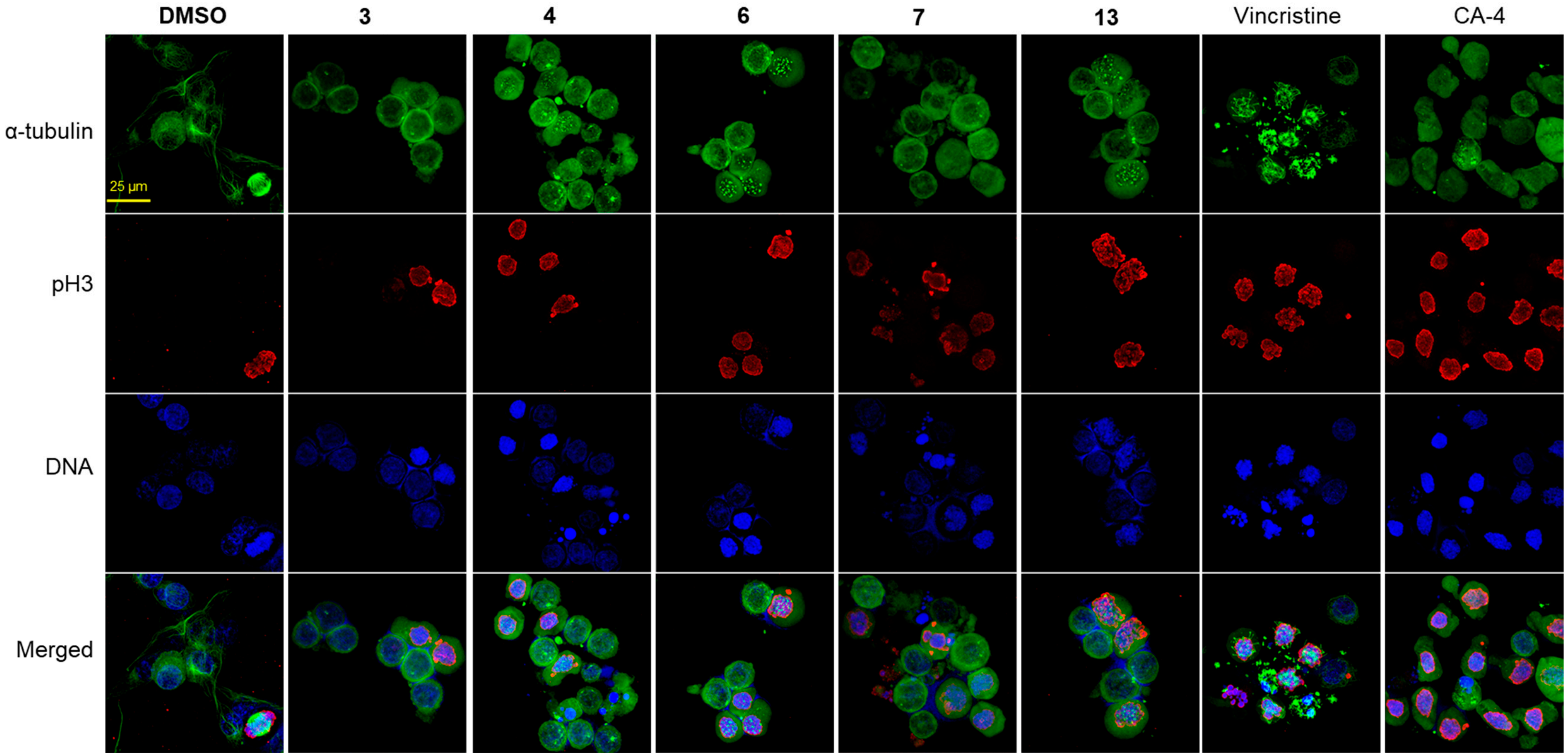

Supplemental Figure S1. Inhibition of tubulin polymerization by compounds.

Vincristine-resistant KB-VIN cells were treated with compounds for 24 h at a concentration of 3× IC_{50}. Fixed cells were stained with antibodies to α-tubulin (green) and phospho-histone H3 (pH3, red), and DAPI was used for DNA (blue). Stained cells were observed by confocal fluorescence microscope, and confocal images were reconstructed. Bar, 0.025 mm.

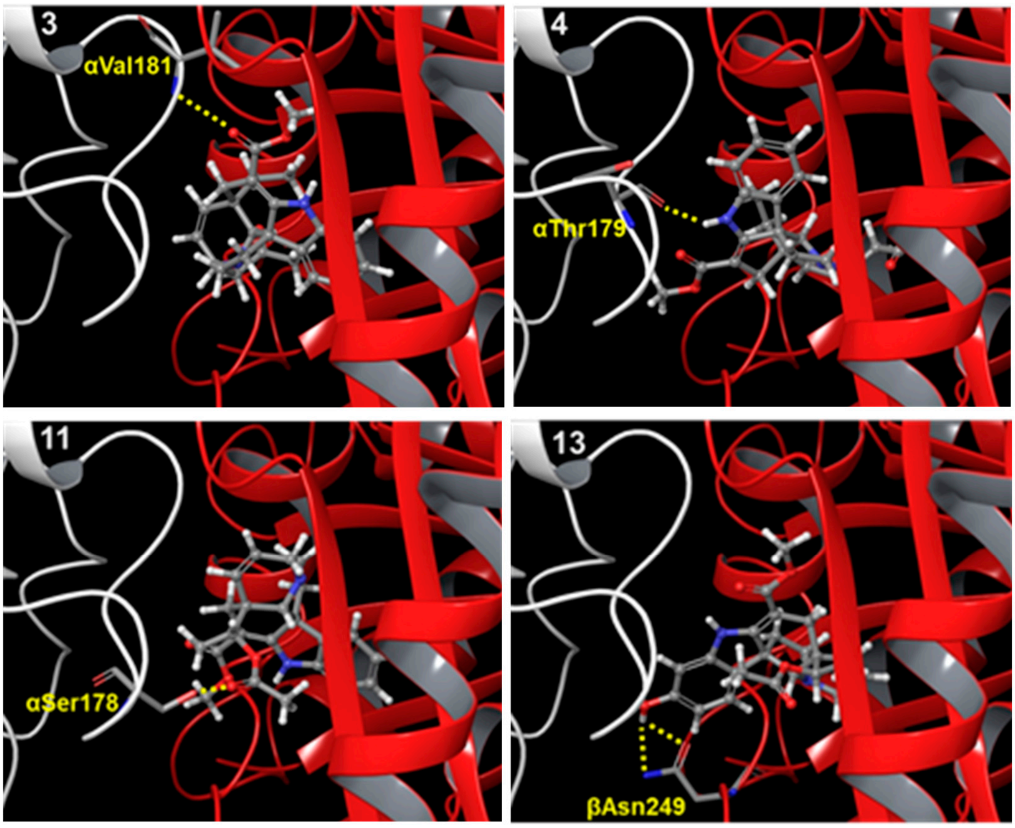

Supplementary Figure S2. Predicted docking models for 3, 4, 11, and 13 binding in the CS.

The crystal structures (PDB: 1SA0) of α- (white) and β-tubulin (red) are shown as ribbon diagrams. The distance calculated to be less than 5 Å between heavy atoms is represented by dashed lines. Docking models of compounds (gray skeleton with oxygen in red and nitrogen in blue) **3**, **4**, **11**, and **13** in the CS are shown. Superimposition of docked compounds with the specific amino acid residue forming H-bonds (yellow dashed line) are shown.

Table S1. ^{1}H NMR data (δ) for compounds **1**-**7** (δ_H, J in Hz).

No.	**1**[a]	**2**[a]	**3**[a]	**4**[a]	**5**[a]	**6**[b]	**7**[c]
NH	9.31 (s)	9.20 (s)	9.40 (s)	9.34 (s)	9.31 (s)	8.90 (s)	9.23 (s)
3a	3.42 (ddd, 16.0, 5.0, 1.5)	2.89 (m)	4.00 (m)	4.05 (dd, 13.0, 5.5)	3.50 (dd, 12.5, 5.5)	3.50 (dd, 12.5, 5.5)	3.51 (dd, 12.0, 5.0)
3b	3.18 (dt, 16.0, 1.5)	2.58 (m)			2.79 (d, 12.5)	2.88 (dd, 12.5, 3.5)	2.90 (dd, 12.0, 3.0)
5a	3.00 (t-like, 7.5)	3.10 (m)	3.11 (m)	2.78 (m)	2.85 (dd, 8.5, 6.5)	2.86 (dd, 8.5, 6.5)	2.80 (dd, 8.0, 6.0)
5b	2.73 (m)	2.42 (td, 12.0, 3.0)	2.99 (m)	2.82 (m)	2.48 (br d, 8.5)	2.43 (dd, 8.5, 4.0)	2.45 (dd, 8.5, 4.0)
6a	1.99 (ddd, 12.0, 11.0, 6.5)	1.96 (ddd, 12.0, 11.5, 6.5)	2.03 (m)	1.83 (m)	1.86 (ddd, 12.0, 11.5, 7.0)	1.91 (ddd, 12.0, 11.5, 7.0)	1.97 (m)
6b	1.71 (dd, 11.0, 4.0)	1.60 (12.0, 5.0)	1.90 (m)	1.62 (dd, 11.0, 4.0)	1.60 (dd, 12.0, 4.5)	1.68 (dd, 12.0, 5.0)	1.63 (m)
9	7.29 (d, 7.5)	7.24 (d, 7.5)	7.34 (d, 7.5)	7.21 (d, 7.5)	7.21 (d, 7.5)	7.01 (d, 8.0)	7.01 (d, 8.0)
10	6.85 (td, 7.5, 1.0)	6.83 (td, 7.5, 1.5)	6.86 (t, 7.5)	6.84 (t, 7.5)	6.83 (t, 7.5)	6.37 (dd, 8.0, 2.0)	6.33 (dd, 8.0, 2.0)
11	7.13 (td, 7.5, 1.0)	7.10 (td, 7.5, 1.5)	7.13 (t, 7.5)	7.13 (t, 7.5)	7.11 (t, 7.5)		
12	7.02 (d, 7.5)	7.00 (d, 7.5)	7.03 (d, 7.5)	7.03 (d, 7.5)	7.00 (d, 7.5)	6.40 (d, 2.0)	6.57 (d, 2.0)
14a	5.78 (ddd, 10.0, 5.0, 1.5)	1.81 (m)	5.86 (dd, 10.0, 3.5)	3.56 (t, 4.2)	3.45 (dd, 5.5, 4.0)	3.48 (dd, 5.5, 3.5)	3.40 (dd, 5.0, 3.0)
14b		1.53 (m)					

15a	5.69 (dt, 10.0, 2.0)	1.78 (m)	5.75 (d, 10.0)	3.15 (d, 4.2)	3.06 (d, 4.0)	3.10 (d, 3.5)	3.06 (d, 3.0)
15b		1.26 (m)					
17a	2.53 (dd, 14.0, 2.0)	2.73 (d, 13.0)	2.59 (d, 12.5)	2.57 (d, 14.5)	2.55 (dd, 15.0, 2.0)	2.56 (dd, 15.0, 2.0)	2.54 (d, 15.0)
17b	2.47 (d, 14.0)	2.26 (dd, 13.0, 2.0)	2.17 (d, 12.5)	2.36 (d, 14.5)	2.48 (dd, 15.0, 5.0)	2.47 (d, 15.0, 5.0)	2.45 (d, 15.0)
18	0.62 (t, 7.5)	0.57 (t, 7.5)	0.66 (t, 7.5)	0.72 (t, 7.5)	0.70 (t, 7.5)	0.74 (t, 7.5)	0.72 (t, 8.0)
19a	0.98 (m)	0.96 (m)	1.03 (m)	1.03 (m)	1.03 (m)	1.13 (m)	1.05 (m)
19b	0.82 (m)	0.62 (m)	0.90 (m)	0.84 (m)	0.83 (m)	0.90 (m)	0.90 (m)
21	2.68 (br s)	2.48 (s)	3.10 (br s)	2.73 (s)	2.40 (br s)	2.40 (br s)	2.34 (br s)
CO_2CH_3	3.69 (s)	3.68 (s)	3.69 (s)	3.70 (s)	3.71 (s)	3.77 (s)	3.70 (s)
11-OCH_3						3.79 (s)	
CH_2COCH_3			2.86 (dd, 13.0, 6.5)	2.96 (m)			
			2.67 (dd, 13.0, 5.5)	2.96 (m)			
CH_2COCH_3			2.21 (s)	2.23 (s)			

[a]500 MHz, acetone-d_6; [b]500 MHz, $CDCl_3$; [c]400 MHz, acetone-d_6.

Table S2. ^{1}H NMR data (δ) for compounds **8**-**14** (δ_H, J in Hz).

No.	**8**[a]	**9**[a]	**10**[b]	**11**[a]	**12**[a]	**13**[b]	**14**[b]
NH	9.30 (s)	9.27 (s)	8.92 (s)	9.33 (s)	9.29 (s)	9.12 (s)	8.95 (s)
3a	3.44 (br d, 13.0)	3.41 (br d, 13.0)	3.48 (ddd, 16.5, 5.0, 1.5)	3.46 (ddd, 16.0, 4.5, 1.5)	3.41 (ddd, 16.0, 5.0, 2.0)	3.49 (ddd, 13.5, 4.0, 1.0)	3.46 (ddd, 16.0, 5.5, 1.5)
3b	2.90 (br d, 13.0)	2.93 (br d, 13.0)	3.26 (dd, 16.5, 1.5)	3.25 (dd, 16.0, 1.5)	3.16 (dt, 16.0, 2.0)	3.25 (dd, 13.5 1.0)	3.20 (dt, 15.0 1.5)
5a	2.93 (m)	3.02 (m)	3.08 (ddd, 9.0, 7.0, 1.5)	3.03 (ddd, 9.5, 7.5, 1.5)	2.98 (t-like, 7.5)	3.07 (ddd, 8.0, 6.0, 1.5)	3.03 (ddd, 8.0, 6.0, 1.5)
5b	2.74 (11.0, 8.5, 5.0)	2.73 (m)	2.78 (ddd, 9.0, 6.0, 2.0)	2.78 (m)	2.70 (ddd, 11.0, 6.5, 4.5)	2.75 (m)	2.71 (m)
6a	1.94 (ddd, 11.5, 11.0, 7.0)	2.01 (ddd, 11.0, 10.0, 6.5)	2.14 (ddd, 12.0, 10.0, 6.0)	2.01 (dd, 11.0, 6.5)	1.97 (12.0, 11.0, 6.5)	2.09 (ddd, 10.0, 9.0, 5.5)	2.05 (ddd, 12.0, 11.0, 6.5)
6b	1.62 (dd, 11.5, 5.0)	1.58 (dd, 11.0, 5.0)	1.88 (ddd, 12.0, 6.0, 2.0)	1.75 (ddd, 11.0, 6.0, 1.5)	1.69 (12.0, 4.5, 1.0)	1.85 (ddd, 10.0, 4.5, 1.0)	1.80 (ddd, 12.0, 6.0, 1.5)
9	7.25 (d, 7.5)	7.18 (d, 7.5)	7.29 (d, 7.5)	7.34 (d, 7.5)	7.17 (d, 7.5)	7.07 (d, 7.0)	7.08 (d, 8.5)
10	6.83 (t, 7.5)	6.38 (d, 7.5)	6.91 (td, 7.5, 1.5)	6.86 (td, 7.5, 1.5)	6.40 (dd, 7.5, 2.0)	6.34 (dd, 7.0, 2.0)	6.34 (dd, 8.5, 2.5)
11	7.13 (t, 7.5)		7.18 (td, 7.5, 1.5)	7.15 (td, 7.5, 1.5)			
12	7.02 (d, 7.5)	6.65 (s)	6.85 (d, 7.5)	7.05 (d, 7.5)	6.67 (d, 2.0)	6.40 (d, 2.0)	6.43 (d, 2.5)
14	3.17 (br d, 3.5)	3.19 (br d, 3.5)	5.94 (ddd, 10.5, 5.0, 1.5)	5.95 (ddd, 10.0, 5.0, 1.5)	5.77 (ddd, 10.0, 5.0, 2.0)	5.97 (ddd, 8.5, 4.0, 1.5)	5.93 (ddd, 10.0, 5.0, 1.0)
15	2.96 (br d, 3.5)	2.94 (br d, 3.5)	5.82 (dt, 10.5, 1.5)	5.76 (dt, 10.0, 1.5)	5.68 (dt, 10.0, 2.0)	5.81 (dt, 8.5, 2.0)	5.77 (dt, 10.0, 1.0)

17a	2.56 (br s)	2.54 (br d, 13.0)	2.91 (dd, 15.0, 2.0)	2.79 (d, 17.4)	2.50 (dd, 15.0, 1.5)	2.81 (dd, 15.0, 1.5)	2.77 (dd, 15.0, 2.0)
17b	2.56 (br s)	2.54 (br d, 13.0)	2.52 (d, 15.0)	2.46 (d, 17.4)	2.45 (d, 15.0)	2.45 (d, 12.5)	2.42 (d, 15.0)
18	0.71 (t, 7.5)	0.72 (t, 7.5)	0.90 (d, 7.0)	0.90 (d, 6.5)	0.62 (t, 7.5)	0.95 (d, 5.0)	0.92 (d, 6.5)
19a	0.92 (q, 7.5)	1.01 (m)	3.36 (q, 7.0)	4.51 (q, 6.5)	0.98 (m)	4.63 (q, 5.0)	4.56 (q, 6.5)
19b	0.92 (q, 7.5)	0.89 (m)			0.82 (m)		
21	2.51 (s)	2.50 (s)	2.80 (s)	2.85 (s)	2.62 (br s)	2.76 (s)	2.73 (s)
CO_2CH_3	3.70 (s)	3.69 (s)	3.81 (s)	3.65 (s)	3.69 (s)	3.76 (s)	3.73 (s)
11-OCH_3		3.75 (s)			3.74 (s)		3.79 (s)
19-OAc				1.91 (s)		1.93 (s)	

[a]500 MHz, acetone-d_6; [b]500 MHz, $CDCl_3$;

Table S3. ^{13}C NMR data (δ) for compounds **1-14**.

No.	**1**[a]	**2**[a]	**3**[a]	**4**[a]	**5**[a]	**6**[b]	**7**[c]	**8**[a]	**9**[a]	**10**[b]	**11**[a]	**12**[a]	**13**[b]	**14**[b]
2	167.0	167.9	165.9	167.6	167.7	168.2	168.5	165.7	166.3	167.8	167.5	167.5	167.5	167.3
3	51.0	52.1	53.1	50.7	50.1	50.2	50.6	49.7	49.7	51.4	50.6	50.8	50.7	50.5
5	51.4	50.9	51.7	47.8	50.6	50.6	50.9	51.2	51.2	51.5	51.5	50.9	51.3	51.2
6	45.6	45.3	46.2	44.8	44.7	44.8	45.8	45.2	44.3	45.3	44.9	44.8	44.8	44.8
7	56.2	55.5	56.7	55.9	54.9	54.3	55.2	56.0	54.2	55.1	55.6	54.7	55.3	55.2
8	138.8	138.2	139.7	138.4	137.5	129.9	129.5	138.7	129.8	138.6	138.1	130.9	129.8	129.3
9	122.1	121.3	122.8	122.5	121.4	121.8	122.5	122.3	122.2	121.9	121.6	121.9	122.6	122.3
10	121.1	120.6	121.5	121.0	120.7	105.0	107.6	121.2	107.2	121.2	120.5	104.9	107.9	105.2
11	128.3	127.5	128.4	128.5	127.8	160.1	158.5	128.5	160.5	128.3	127.9	160.3	157.6	160.5
12	110.4	109.4	110.3	110.6	109.4	96.9	98.9	110.5	97.0	110.1	109.9	96.9	98.6	97.2
13	144.5	143.3	144.5	144.4	143.0	144.2	145.5	144.5	144.3	143.7	143.6	144.7	145.2	144.8
14	126.2	22.8	128.4	57.2	54.0	54.0	54.2	52.3	52.4	124.9	127.8	124.9	127.8	127.6
15	133.4	33.6	132.8	59.1	57.2	57.3	57.1	56.1	56.6	133.8	129.9	133.5	129.8	129.8
16	92.4	92.6	90.9	90.6	90.6	90.9	90.8	91.4	91.3	92.9	91.9	92.8	92.2	91.8
17	27.5	26.2	30.2	24.3	23.2	23.2	24.0	24.6	23.3	28.8	27.9	26.9	28.3	27.8
18	7.6	7.4	8.0	7.5	7.2	7.2	7.4	7.2	8.7	17.9	14.9	7.8	16.1	15.2
19	29.4	29.3	31.7	25.2	24.4	24.4	25.0	27.1	26.4	66.8	67.2	28.6	67.3	66.8
20	42.0	38.6	38.5	41.7	41.0	41.1	41.8	37.8	37.5	48.1	46.6	41.7	46.4	46.2
21	70.6	72.6	65.2	63.4	67.6	67.7	68.6	71.4	71.2	68.5	71.3	70.6	70.8	70.5
CO_2Me	168.0	169.3	168.6	168.3	168.9	168.9	168.6	168.6	169.2	169.6	169.6	169.6	169.2	169.1
	50.9	51.2	50.9	51.2	51.1	51.1	50.9	50.9	51.4	51.3	51.6	51.5	51.5	51.4
11-OMe	-	-	-	-	-	55.5	-	-	56.0	-	-	55.8	-	56.0

19-OAc	-	-	-	-	-	-	-	-	-	-	171.8	-	171.9	171.5
	-	-	-	-	-	-	-	-	-	-	21.5	-	21.5	21.3
CH_2COCH_3	-	-	43.7	37.8	-	-	-	-	-	-	-	-	-	-
	-	-	207.3	207.7	-	-	-	-	-	-	-	-	-	-
	-	-	30.1	30.4	-	-	-	-	-	-	-	-	-	-

[a] 125 MHz, acetone-d_6; [b] 125 MHz, $CDCl_3$; [c] 100 MHz, acetone-d_6.

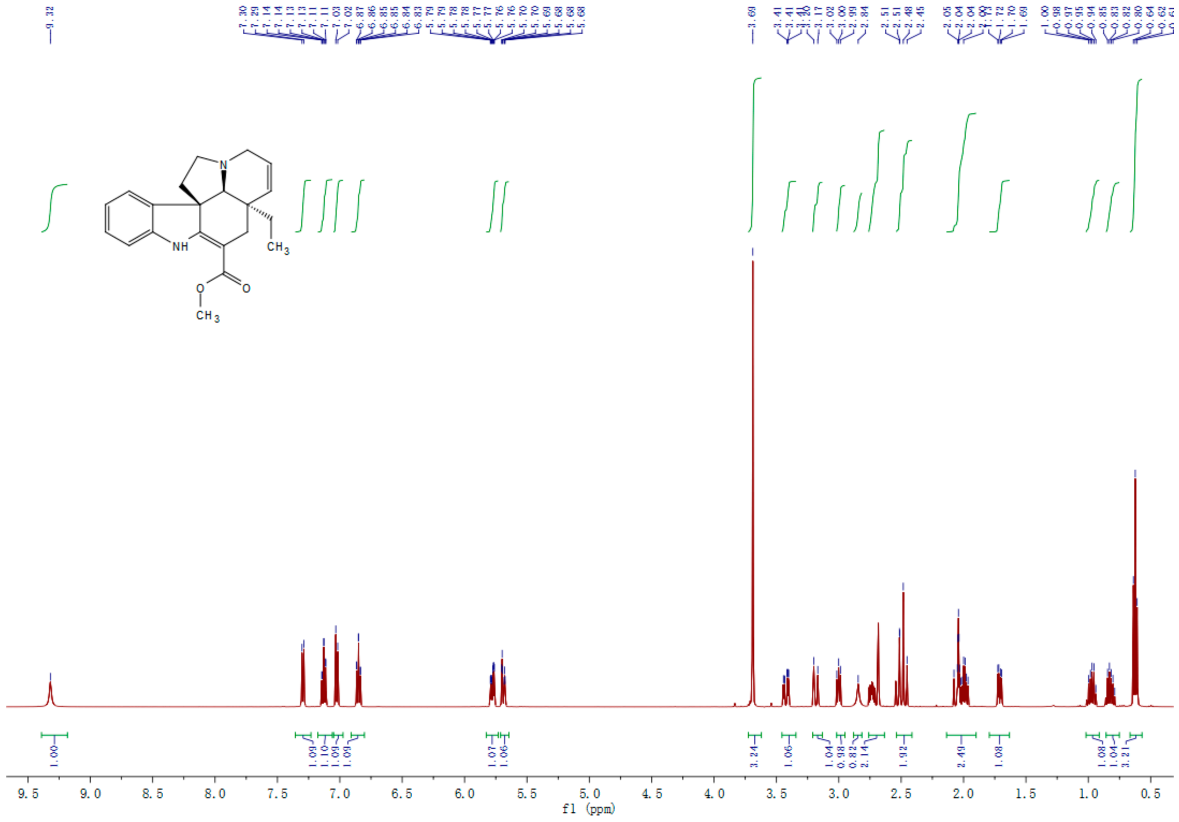
NH
N
CH3
O
O
CH3
fl (ppm)

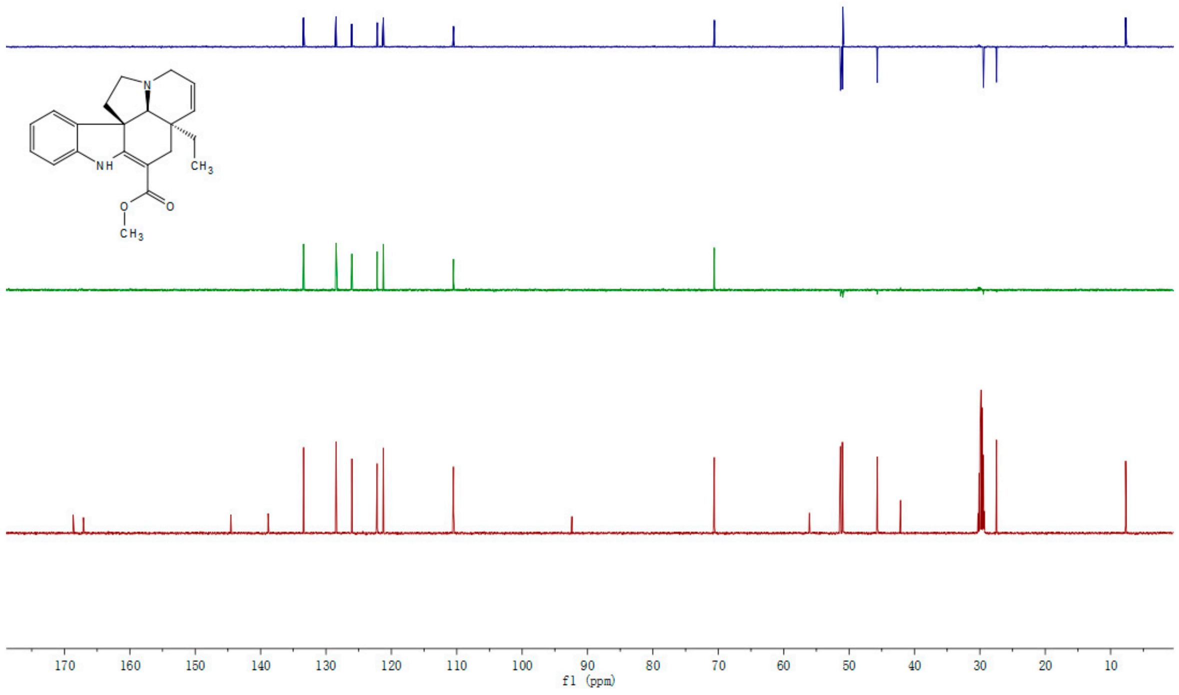
NH
N
CH3
O
O
CH3
fl (ppm)

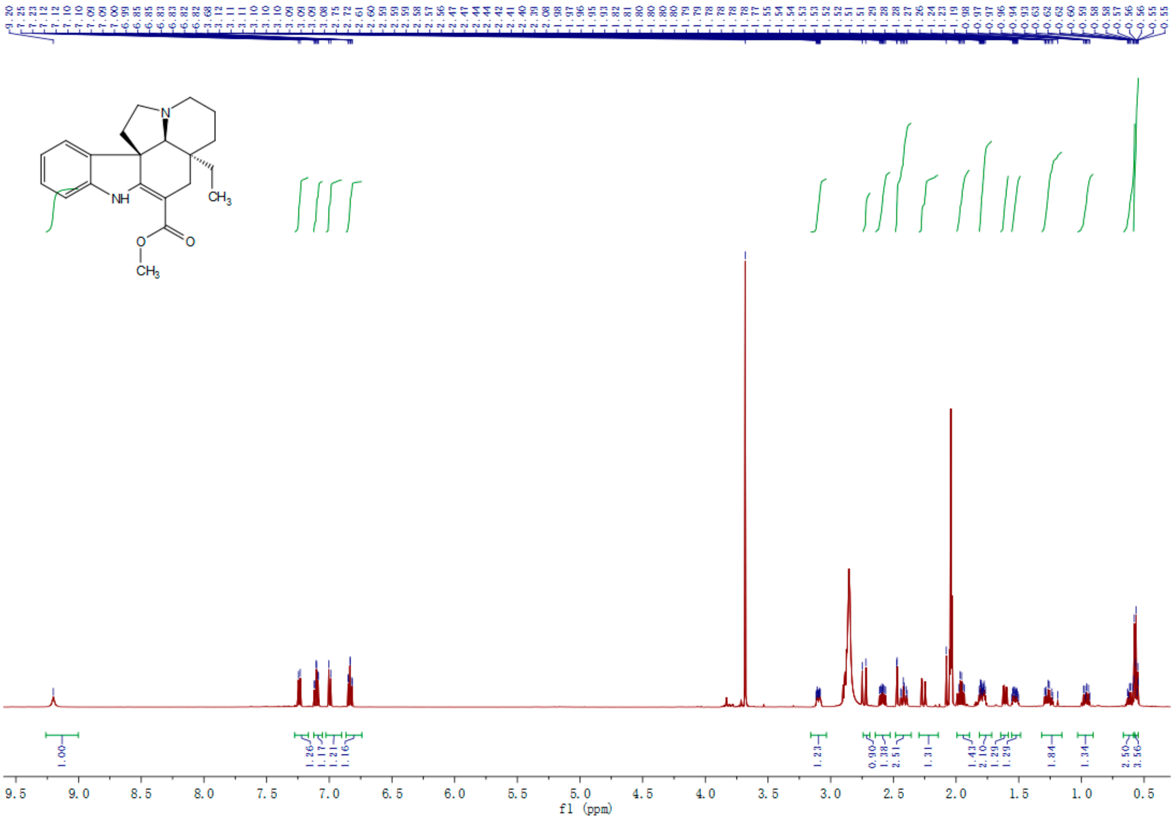

N
NH
CH3
O
O
CH3
9.5 9.0 8.5 8.0 7.5 7.0 6.5 6.0 5.5 5.0 4.5 4.0 3.5 3.0 2.5 2.0 1.5 1.0 0.5
f1 (ppm)

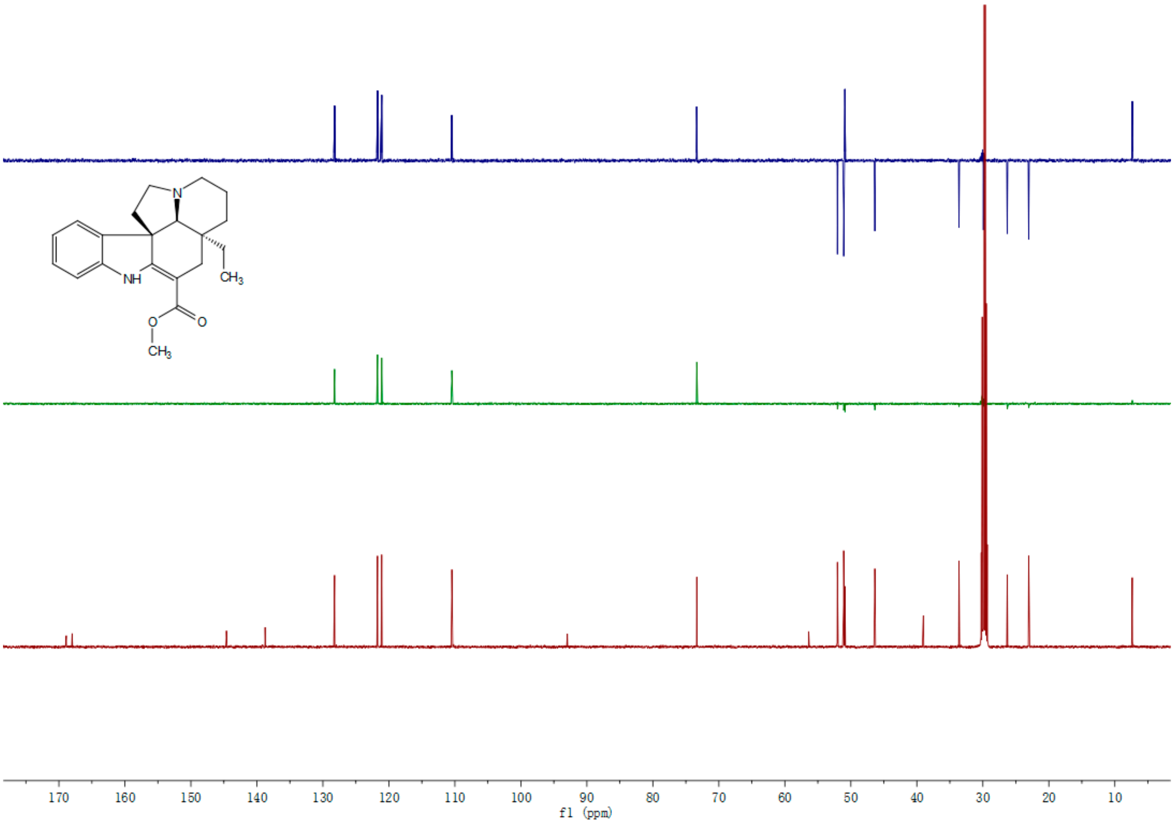

N
NH
CH3
O
O
CH3
170 160 150 140 130 120 110 100 90 80 70 60 50 40 30 20 10
f1 (ppm)

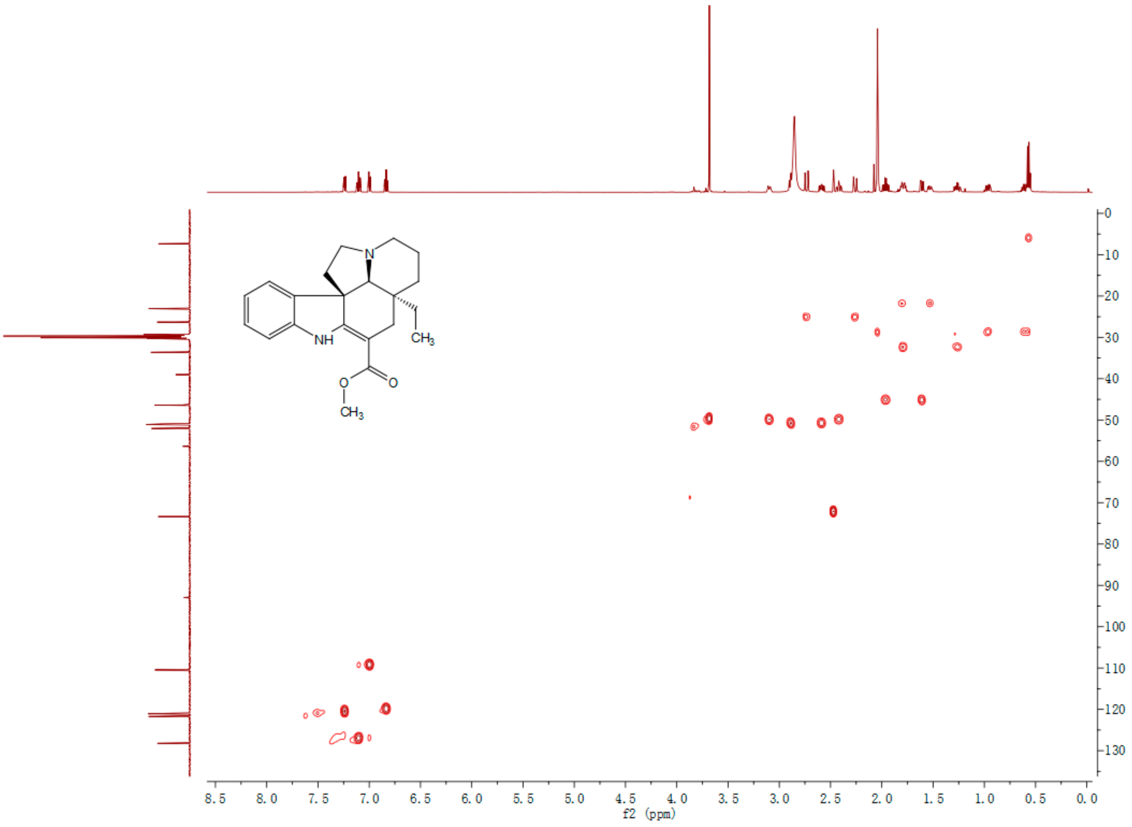
NH
CH3
O
O
CH3
N
f2 (ppm)
8.5 8.0 7.5 7.0 6.5 6.0 5.5 5.0 4.5 4.0 3.5 3.0 2.5 2.0 1.5 1.0 0.5 0.0
0 10 20 30 40 50 60 70 80 90 100 110 120 130

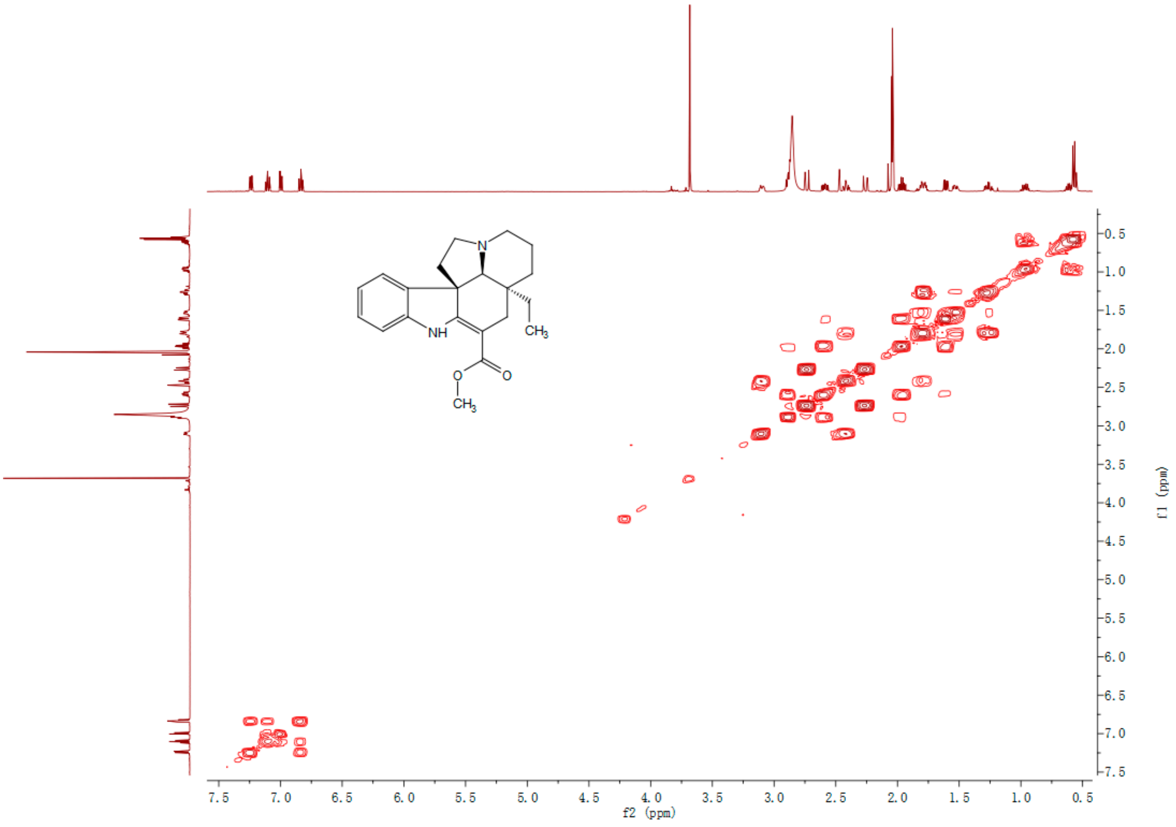
NH
CH3
O
O
CH3
N
f2 (ppm)
f1 (ppm)
7.5 7.0 6.5 6.0 5.5 5.0 4.5 4.0 3.5 3.0 2.5 2.0 1.5 1.0 0.5
0.5 1.0 1.5 2.0 2.5 3.0 3.5 4.0 4.5 5.0 5.5 6.0 6.5 7.0 7.5

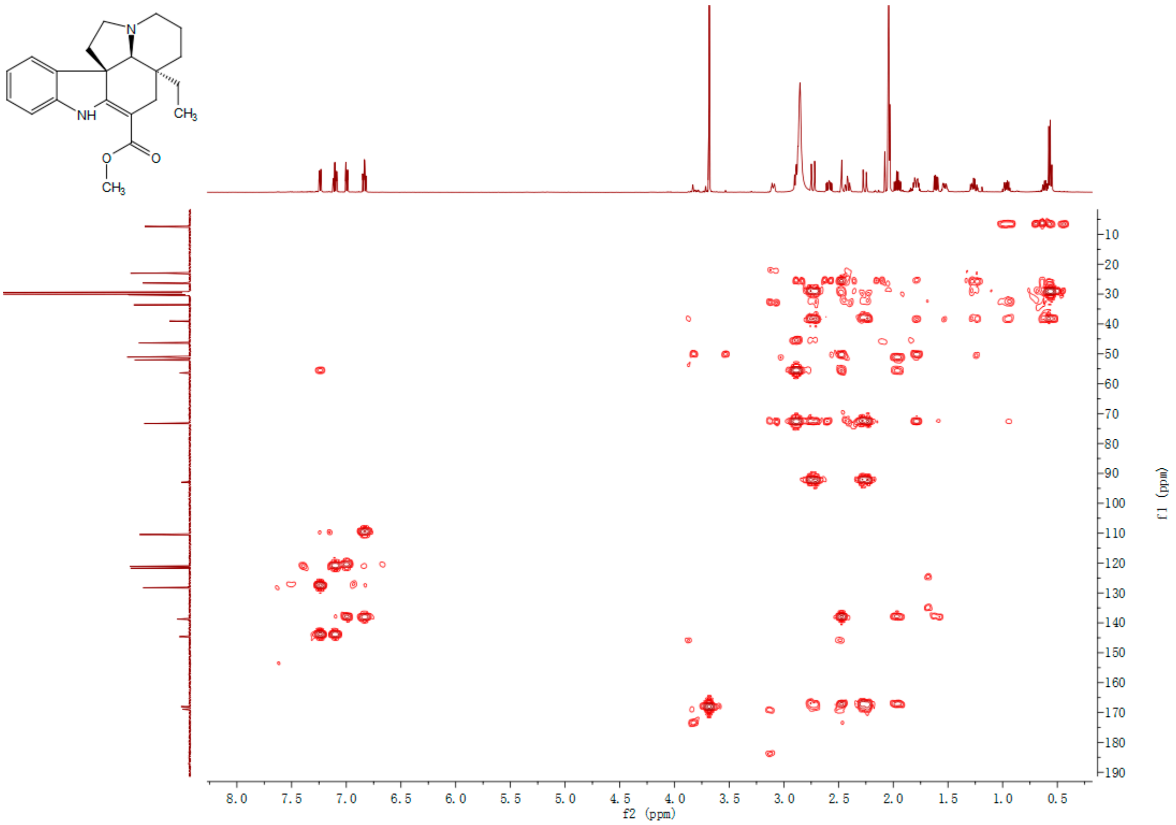
NH
N
CH3
O
O
CH3
f2 (ppm)
f1 (ppm)

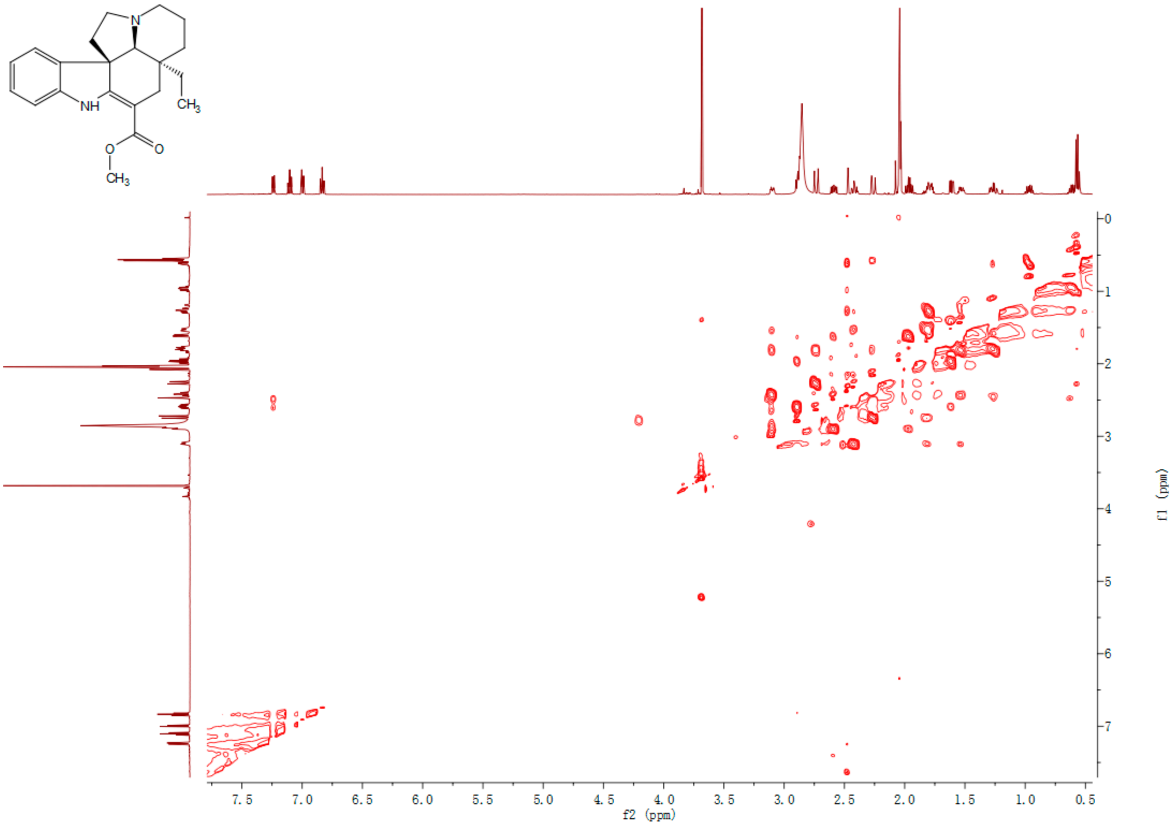
NH
N
CH3
O
O
CH3
f2 (ppm)
f1 (ppm)

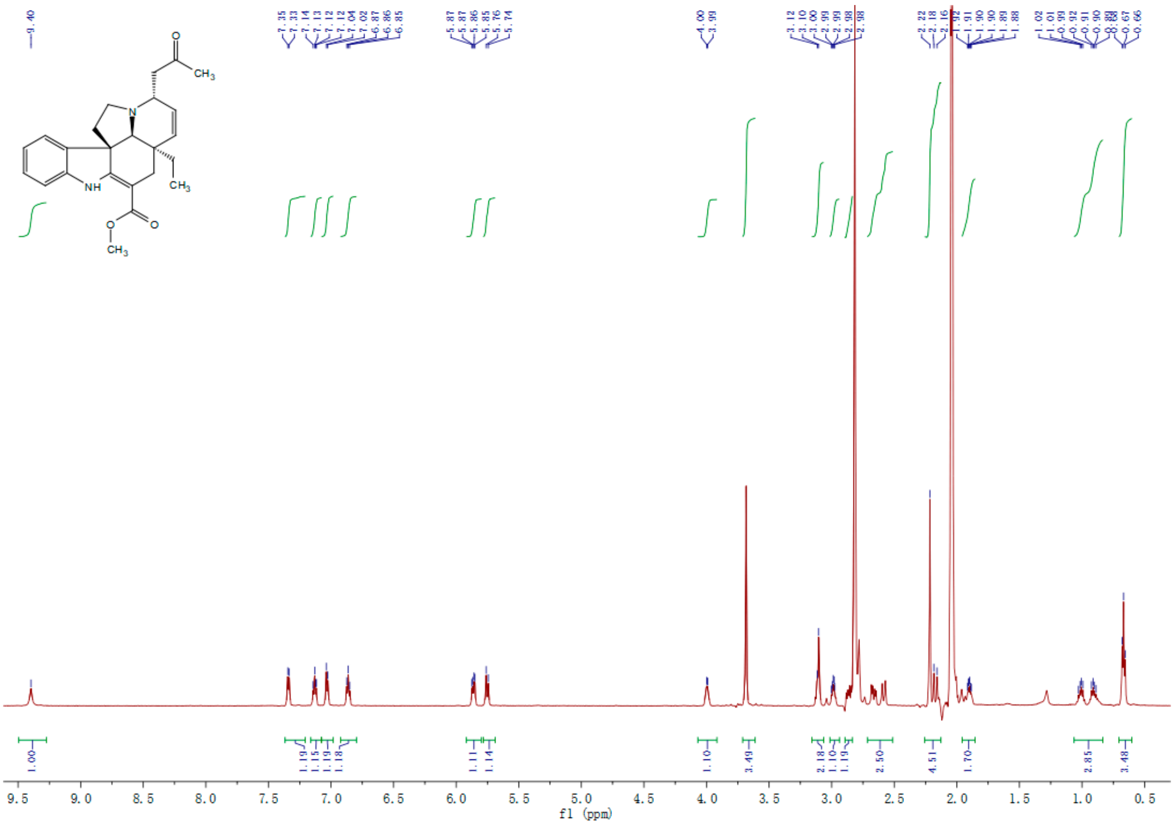

f1 (ppm)

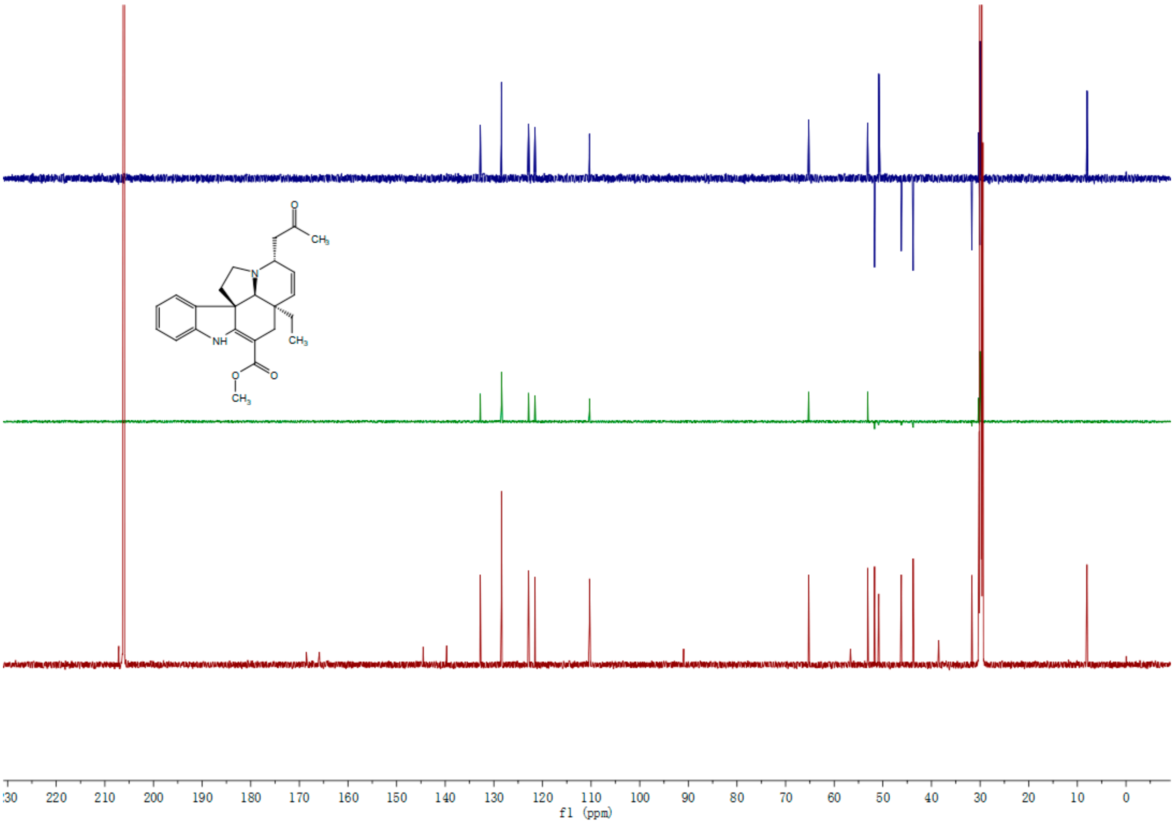

f1 (ppm)

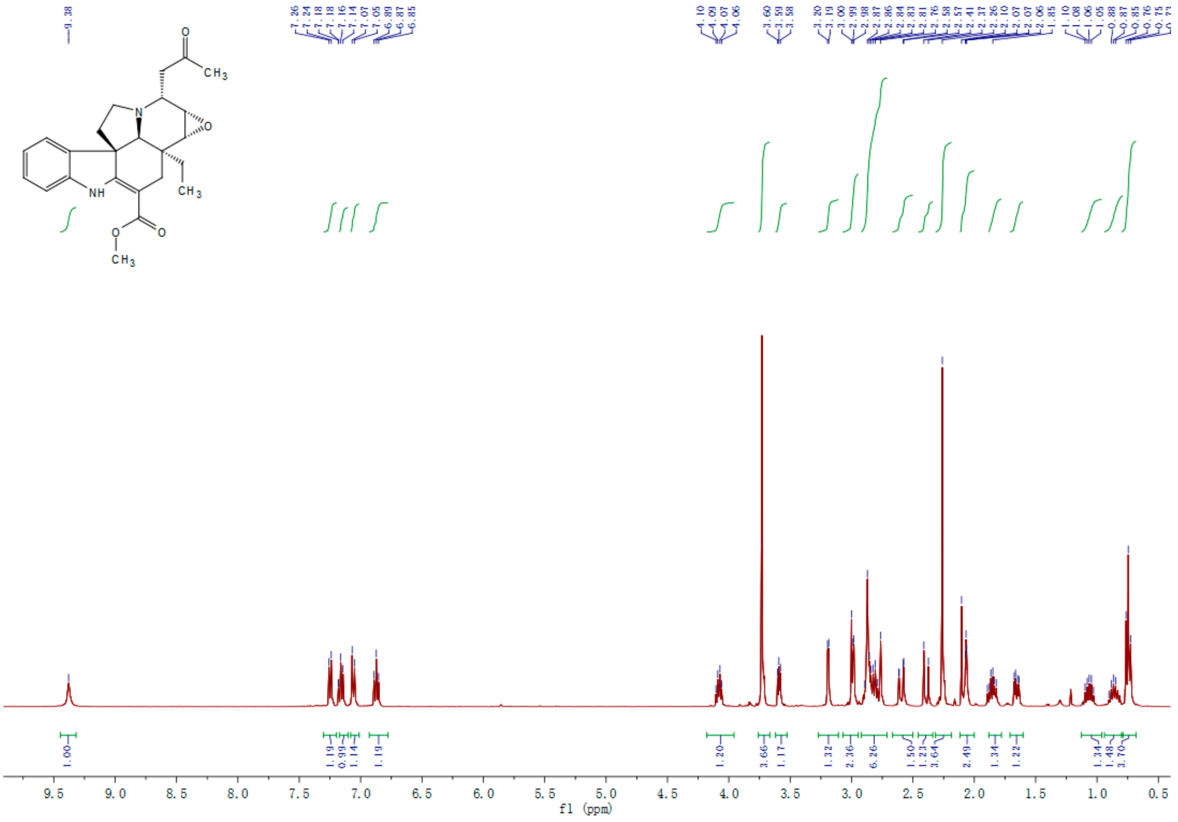

9.38
7.26 7.24 7.18 7.18 7.16 7.14 7.07 7.05 6.89 6.87 6.85
4.10 4.09 4.07 4.06
3.60 3.59 3.58
3.20 3.19 3.00 2.99 2.98 2.87 2.86 2.84 2.83 2.81 2.76 2.58 2.57 2.41 2.37 2.26 2.10 2.07 2.06 1.85
1.10 1.08 1.06 1.05 0.88 0.87 0.85 0.76 0.75 0.73
O
CH3
N
O
NH
CH3
O
O
CH3
1.00
1.19 0.99 1.14 1.19
1.20 3.66 1.17 1.32 2.36 6.26 1.50 1.23 3.64 2.49 1.34 1.22 1.34 1.48 3.70
9.5 9.0 8.5 8.0 7.5 7.0 6.5 6.0 5.5 5.0 4.5 4.0 3.5 3.0 2.5 2.0 1.5 1.0 0.5
f1 (ppm)

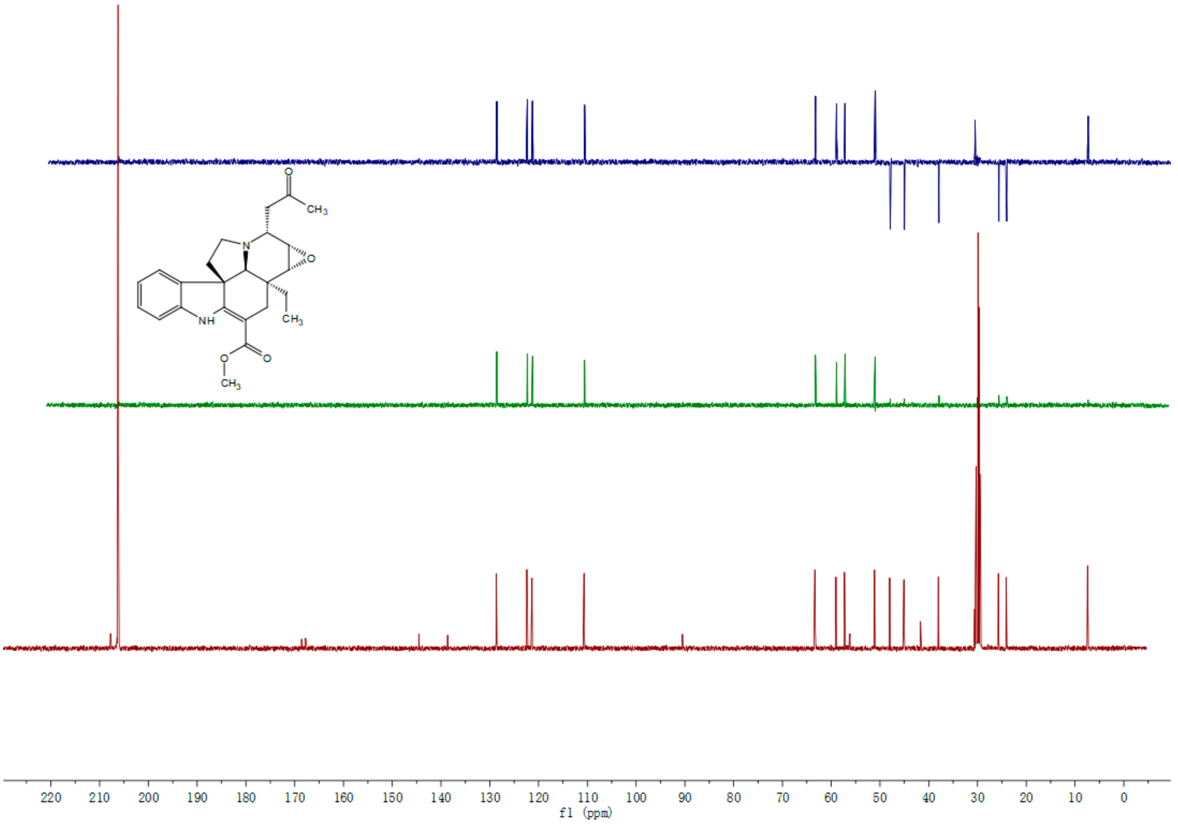

O
CH3
N
O
NH
CH3
O
O
CH3
220 210 200 190 180 170 160 150 140 130 120 110 100 90 80 70 60 50 40 30 20 10 0
f1 (ppm)

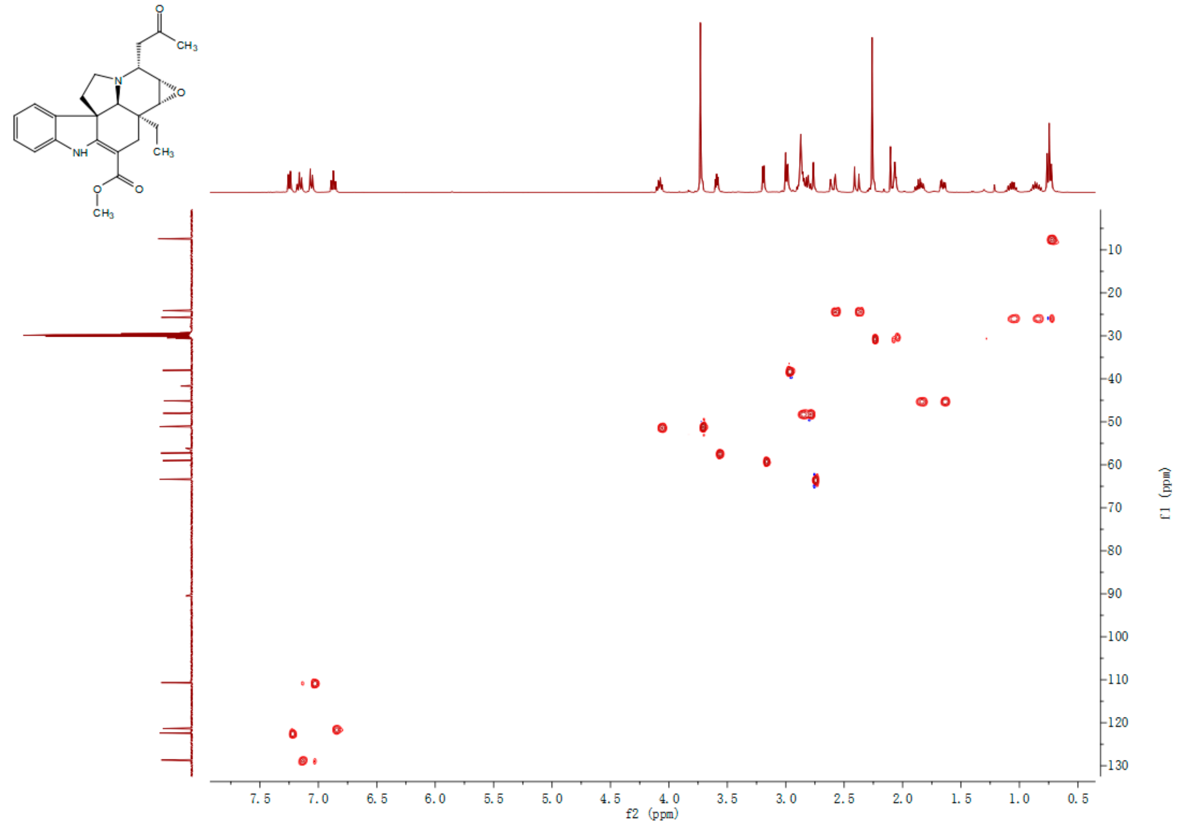

f2 (ppm)
f1 (ppm)

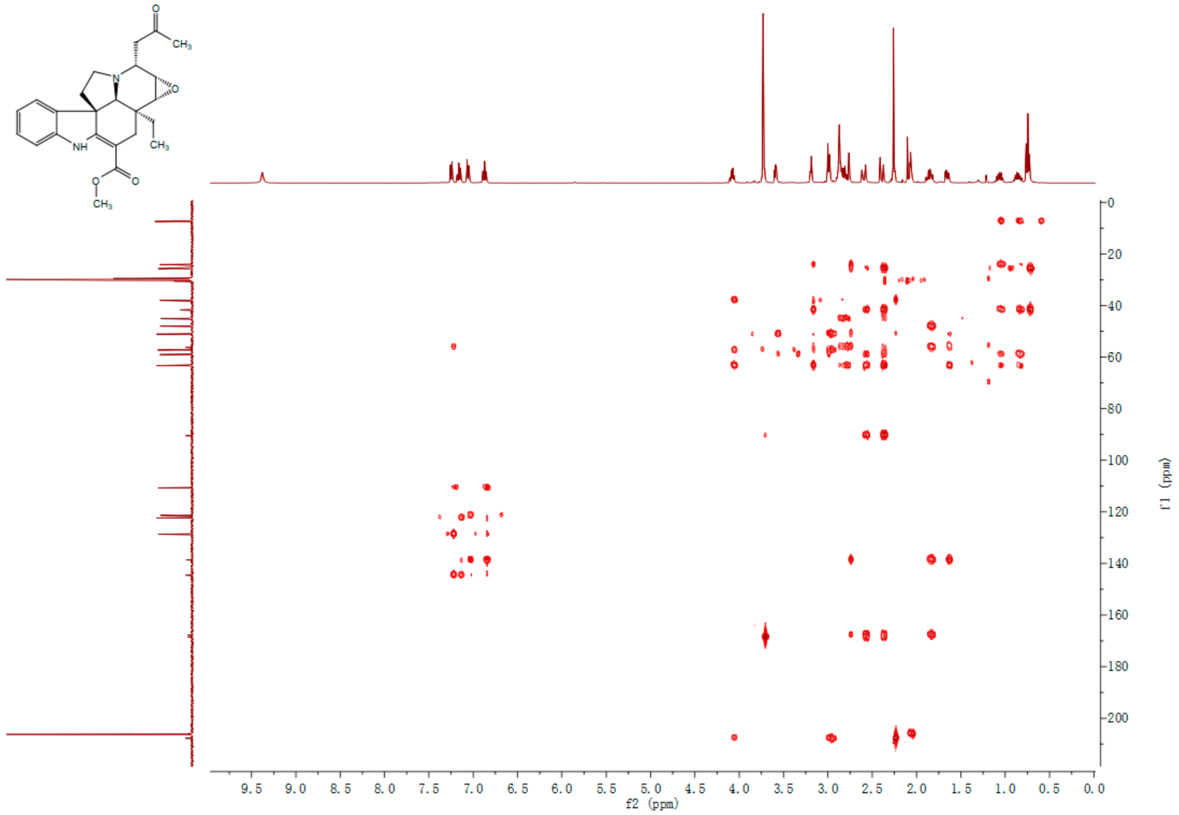

f2 (ppm)
f1 (ppm)

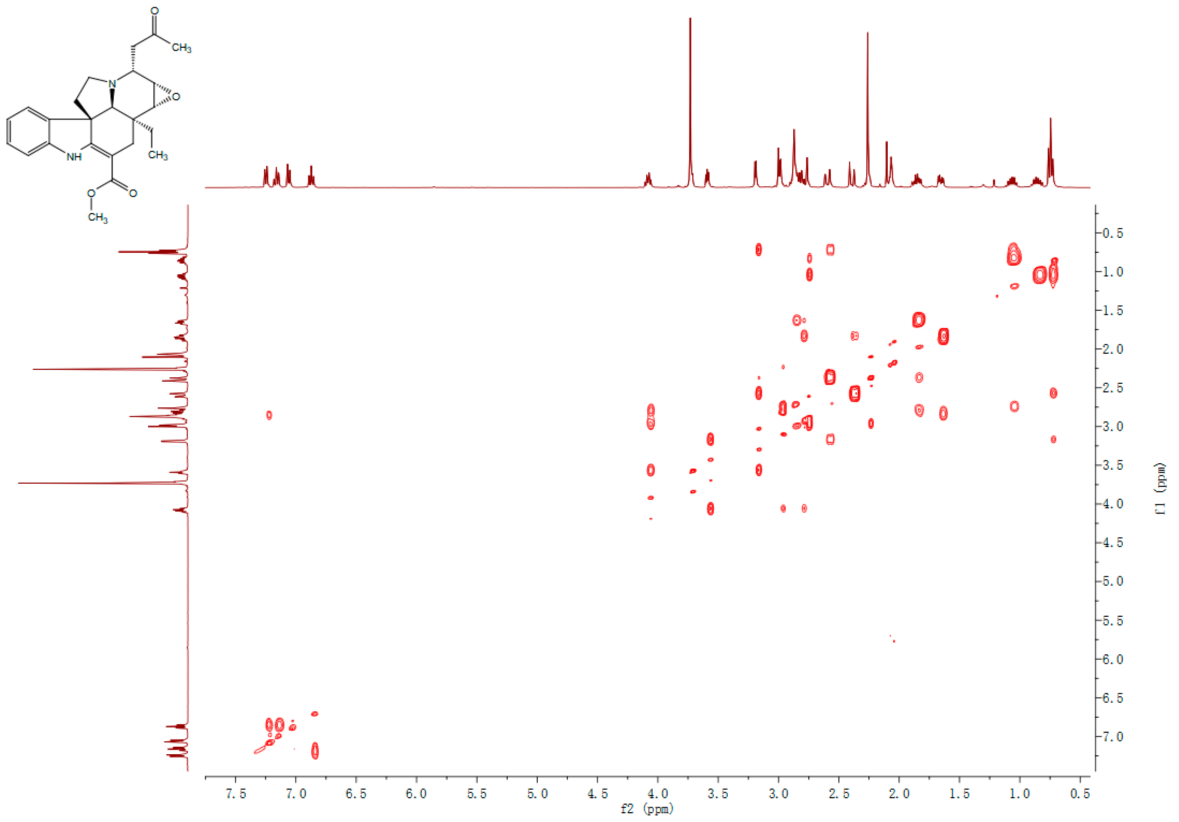

CH3
O
NH
7.5 7.0 6.5 6.0 5.5 5.0 4.5 4.0 3.5 3.0 2.5 2.0 1.5 1.0 0.5
f2 (ppm)
0.5 1.0 1.5 2.0 2.5 3.0 3.5 4.0 4.5 5.0 5.5 6.0 6.5 7.0
f1 (ppm)

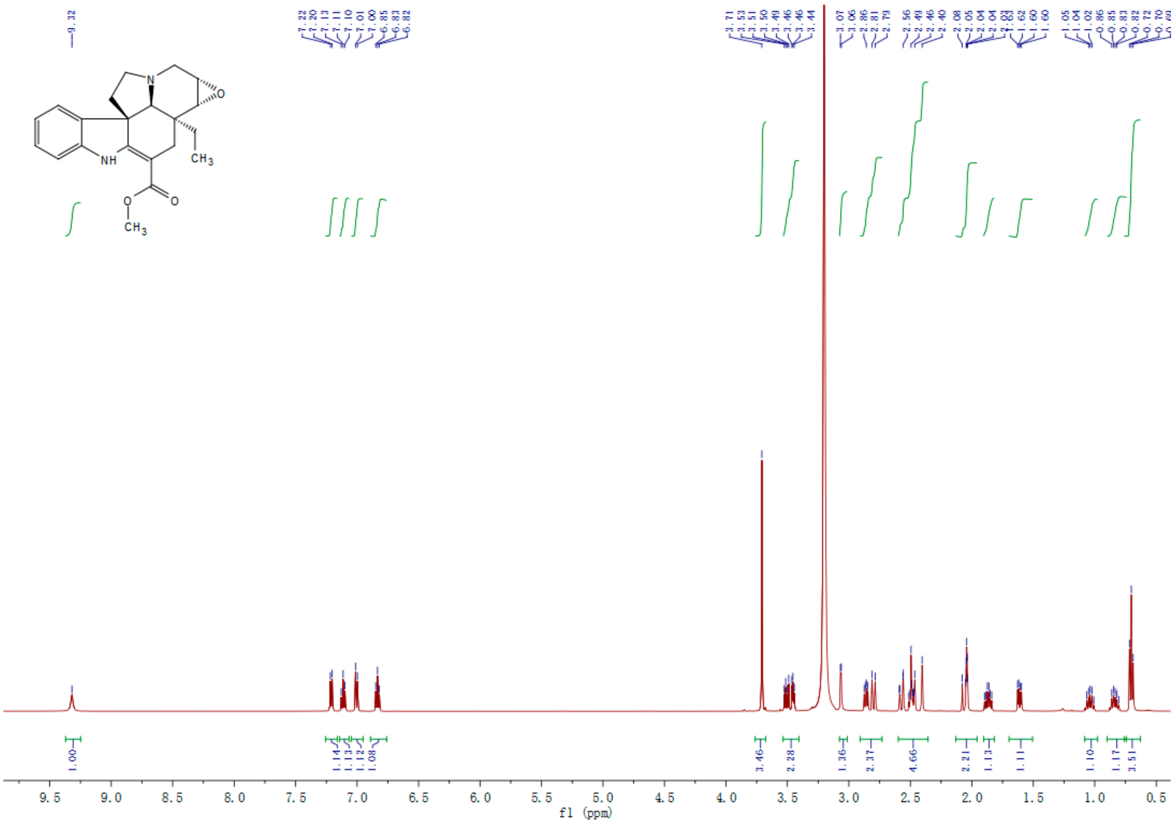

f1 (ppm)

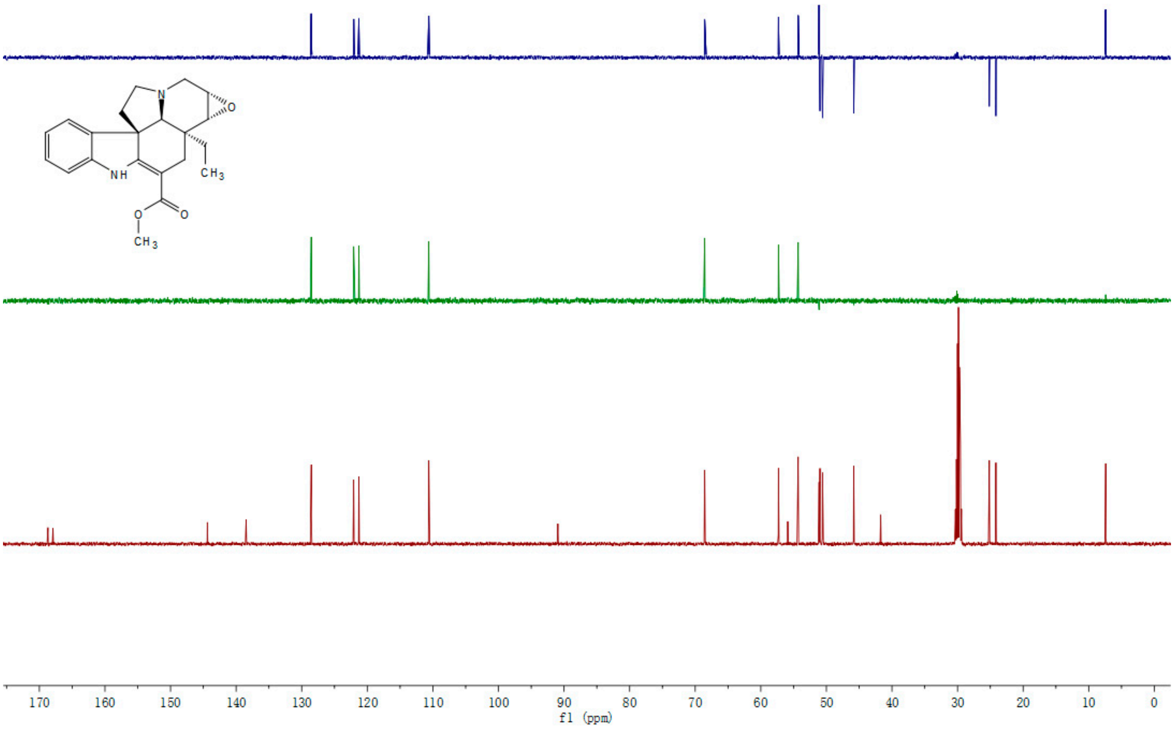

f1 (ppm)

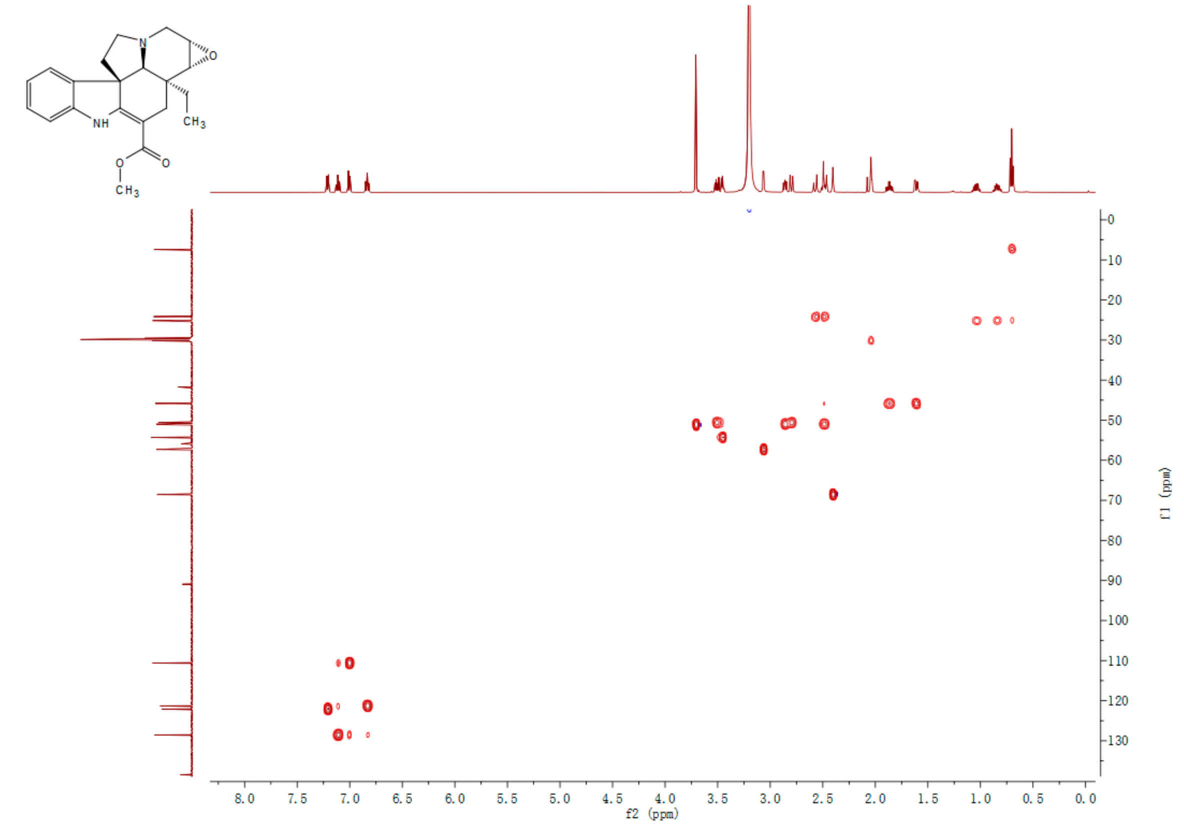

N
O
NH
CH3
O
O
CH3
f2 (ppm)
f1 (ppm)

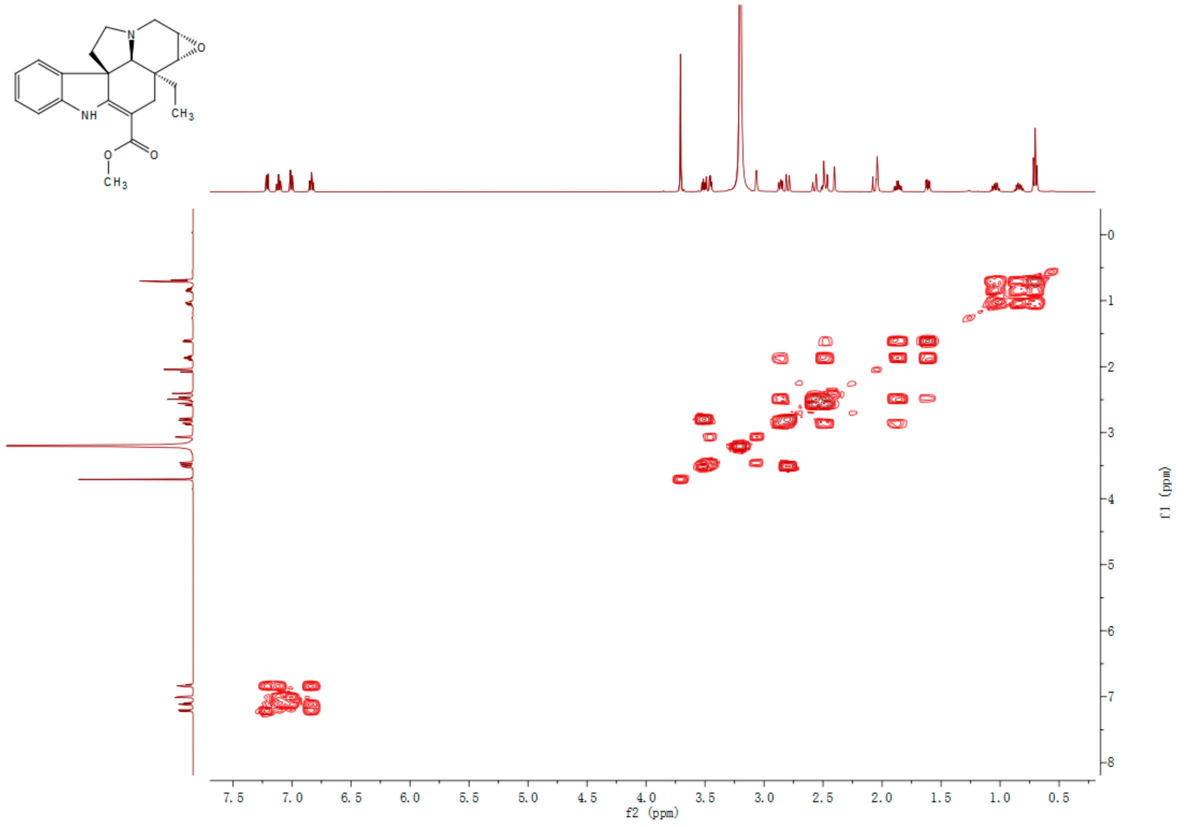

N
O
NH
CH3
O
O
CH3
f2 (ppm)
f1 (ppm)

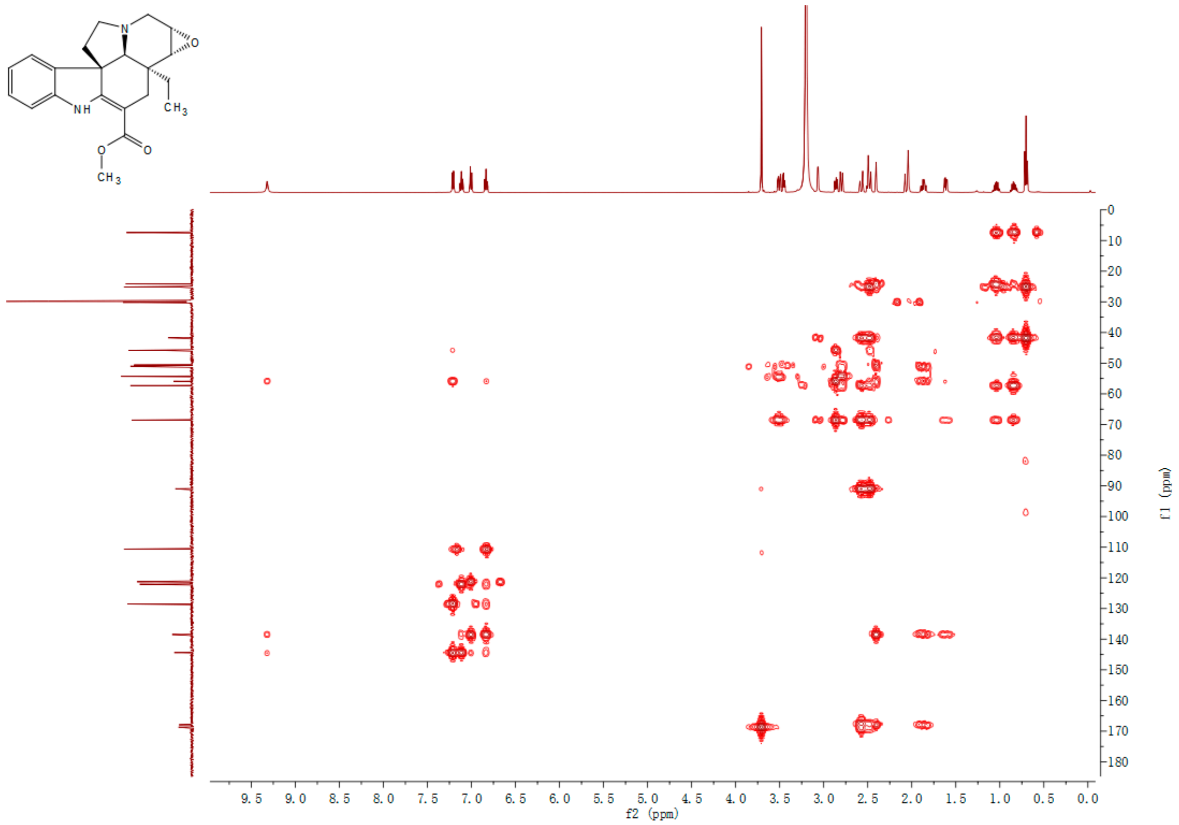

N
O
NH
CH3
O
O
CH3
f2 (ppm)
f1 (ppm)

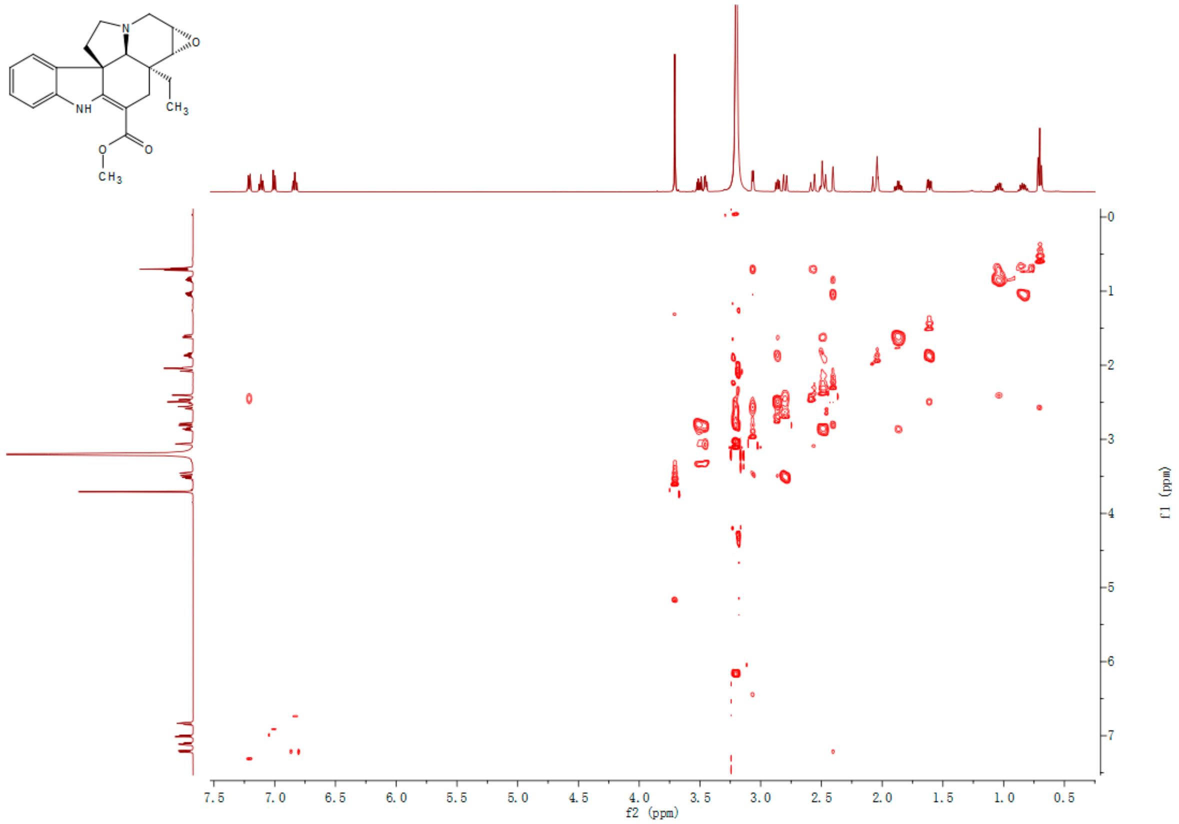

N
O
NH
CH3
O
O
CH3
f2 (ppm)
f1 (ppm)

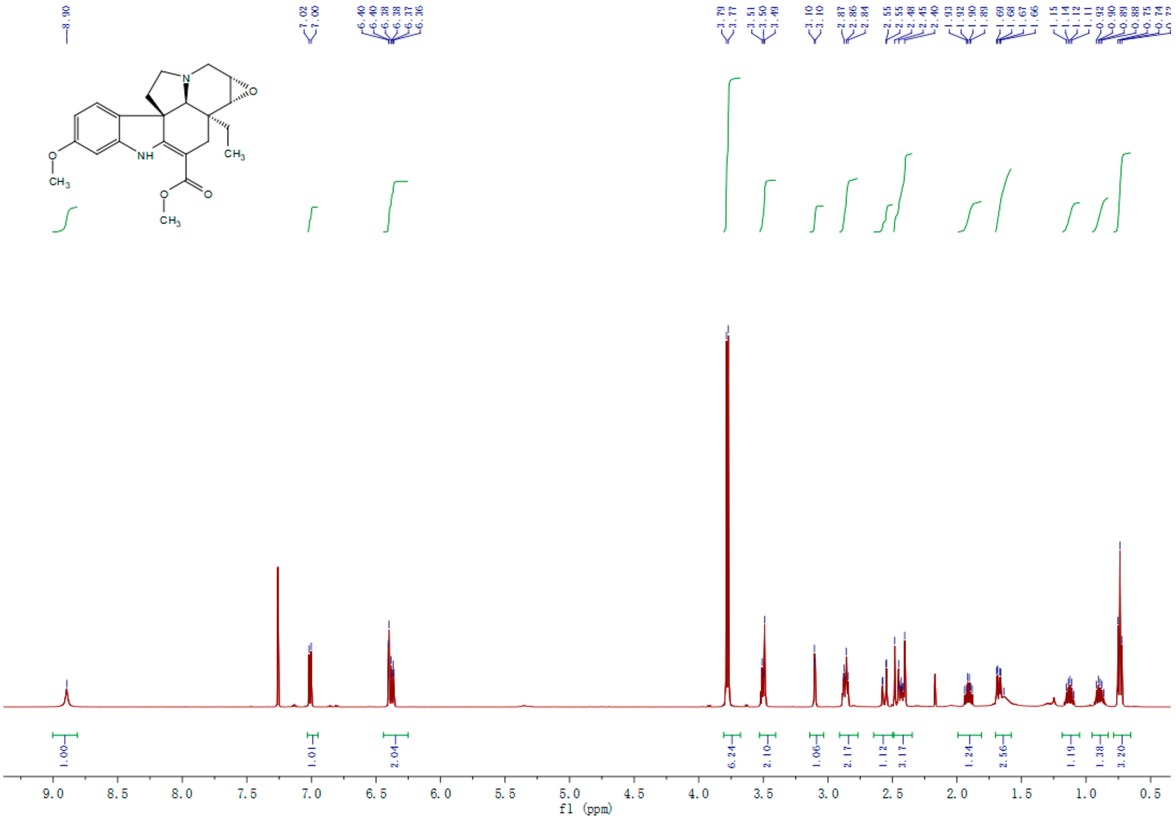
NH
O
N
CH3
1.00
1.01
2.04
6.24
2.10
1.06
2.17
1.12
3.17
1.24
2.56
1.19
1.38
3.20
9.0 8.5 8.0 7.5 7.0 6.5 6.0 5.5 5.0 4.5 4.0 3.5 3.0 2.5 2.0 1.5 1.0 0.5
f1 (ppm)

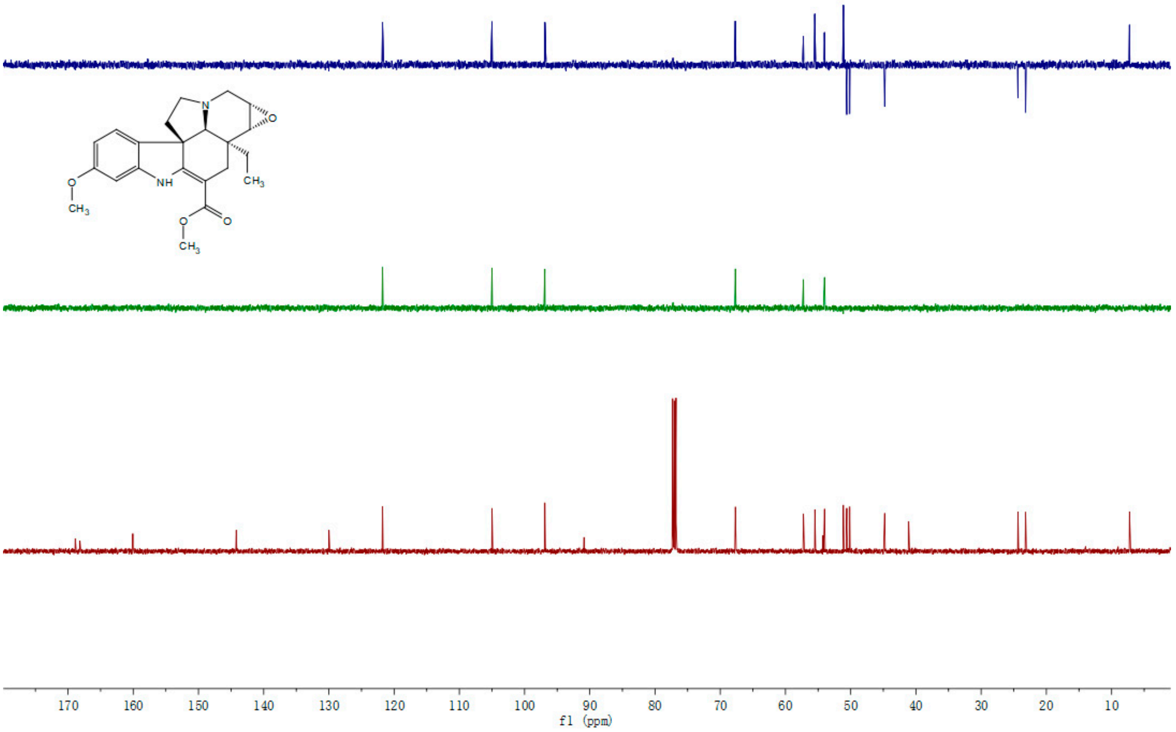
NH
O
N
CH3
170 160 150 140 130 120 110 100 90 80 70 60 50 40 30 20 10
f1 (ppm)

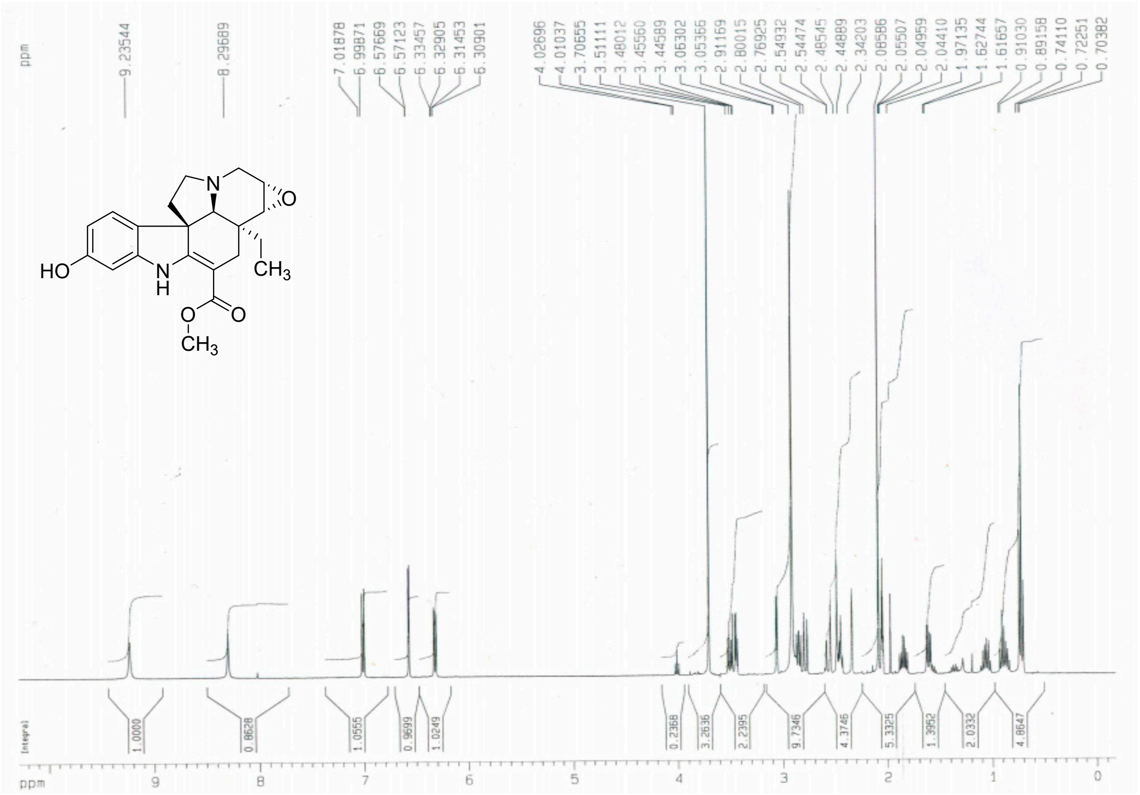

ppm
9.23544
8.29689
7.01878
6.99871
6.57669
6.57123
6.33457
6.32905
6.31453
6.30901
4.02696
4.01037
3.70655
3.51111
3.48012
3.45560
3.44589
3.06302
3.05356
2.91169
2.80015
2.76925
2.54932
2.54474
2.48545
2.44889
2.34203
2.08586
2.05507
2.04959
2.04410
1.97135
1.62744
1.61657
0.91030
0.89158
0.74110
0.72251
0.70382
HO
N
H
N
O
CH3
O
O
CH3
Integral
1.0000
0.8628
1.0555
0.9699
1.0249
0.2368
3.2636
2.2395
9.7346
4.3746
5.3325
1.3952
2.0332
4.8647
ppm
9
8
7
6
5
4
3
2
1
0

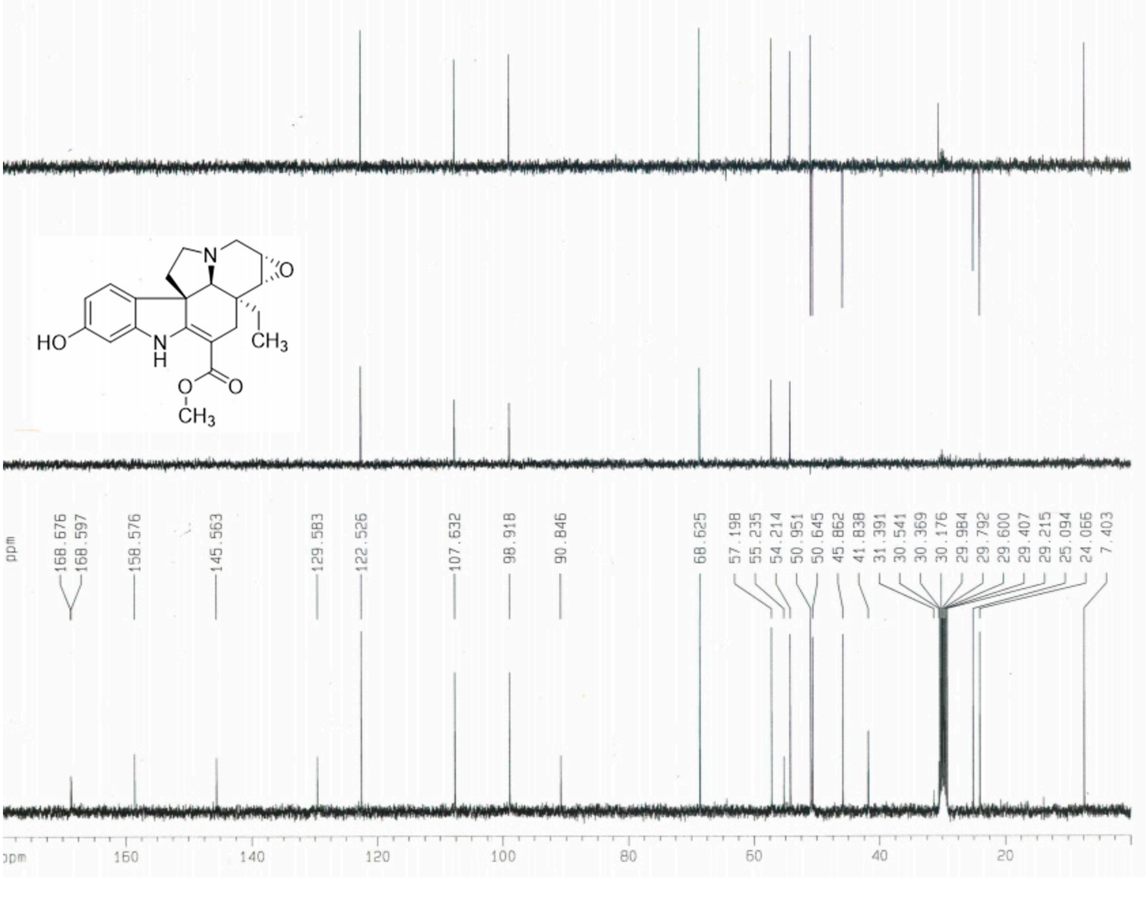

HO
N
H
N
O
CH3
O
O
CH3
ppm
168.676
168.597
158.576
145.563
129.583
122.526
107.632
98.918
90.846
68.625
57.198
55.235
54.214
50.951
50.645
45.862
41.838
31.391
30.541
30.369
30.176
29.984
29.792
29.600
29.407
29.215
25.094
24.066
7.403
ppm
160
140
120
100
80
60
40
20

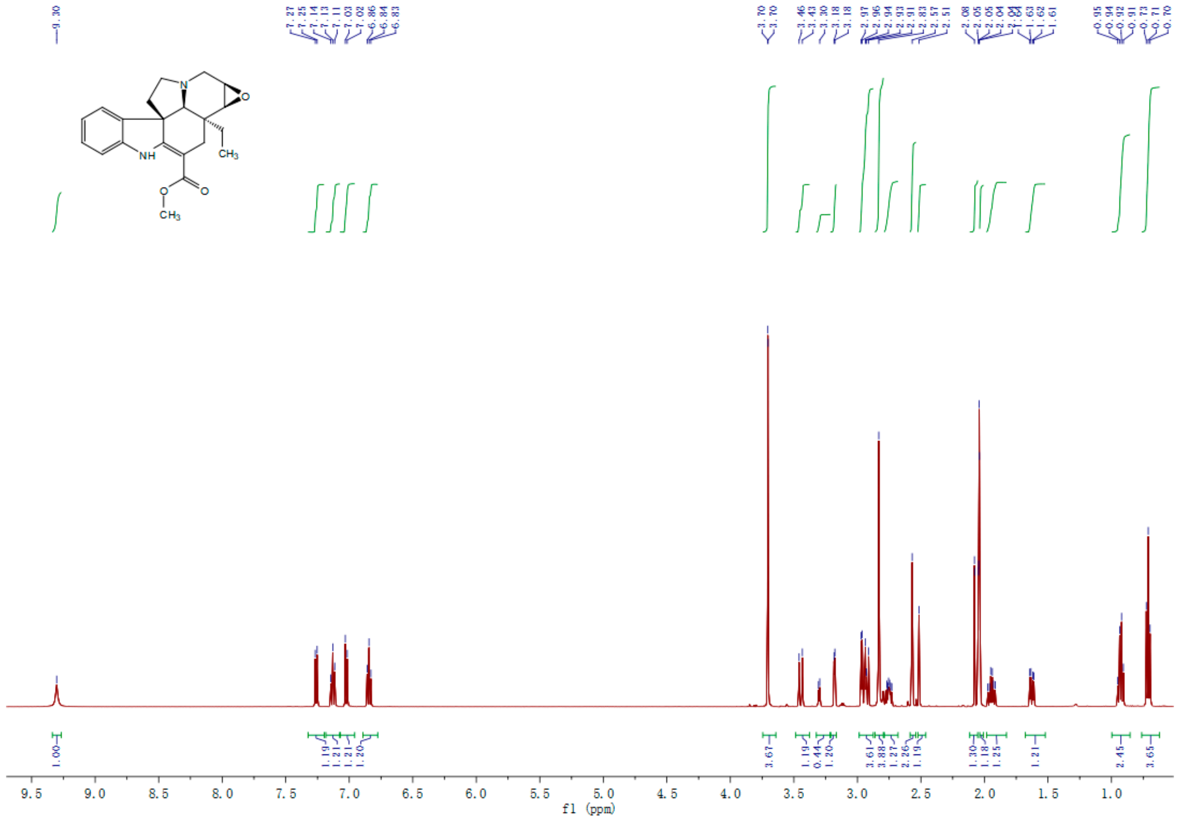

9.5
9.0
8.5
8.0
7.5
7.0
6.5
6.0
5.5
5.0
4.5
4.0
3.5
3.0
2.5
2.0
1.5
1.0
f1 (ppm)

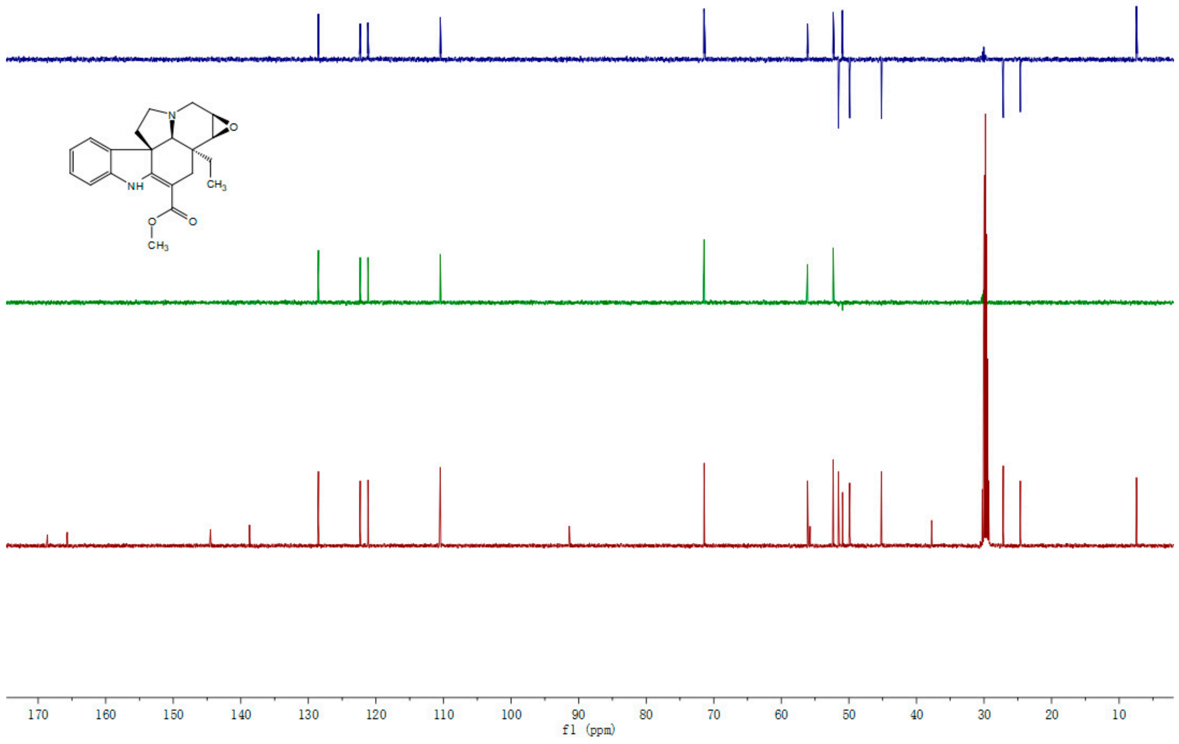

170
160
150
140
130
120
110
100
90
80
70
60
50
40
30
20
10
f1 (ppm)

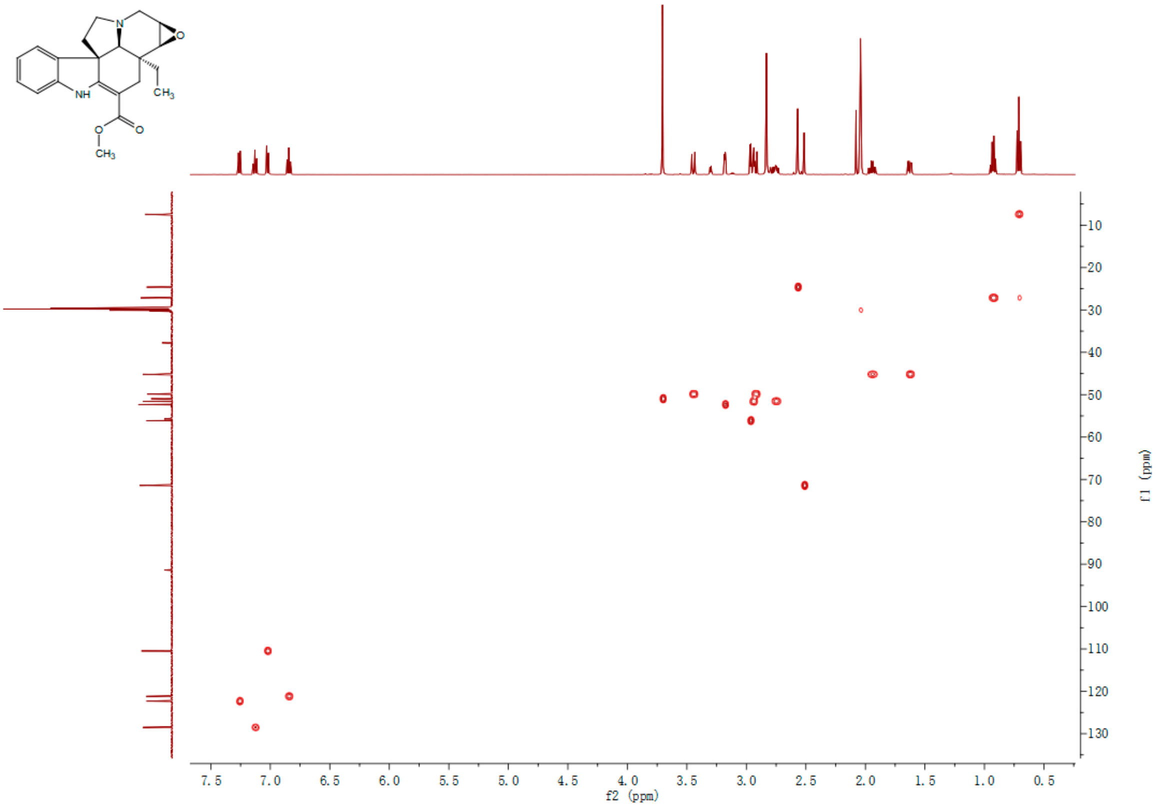
N
O
NH
CH3
O
O
CH3
f2 (ppm)
f1 (ppm)

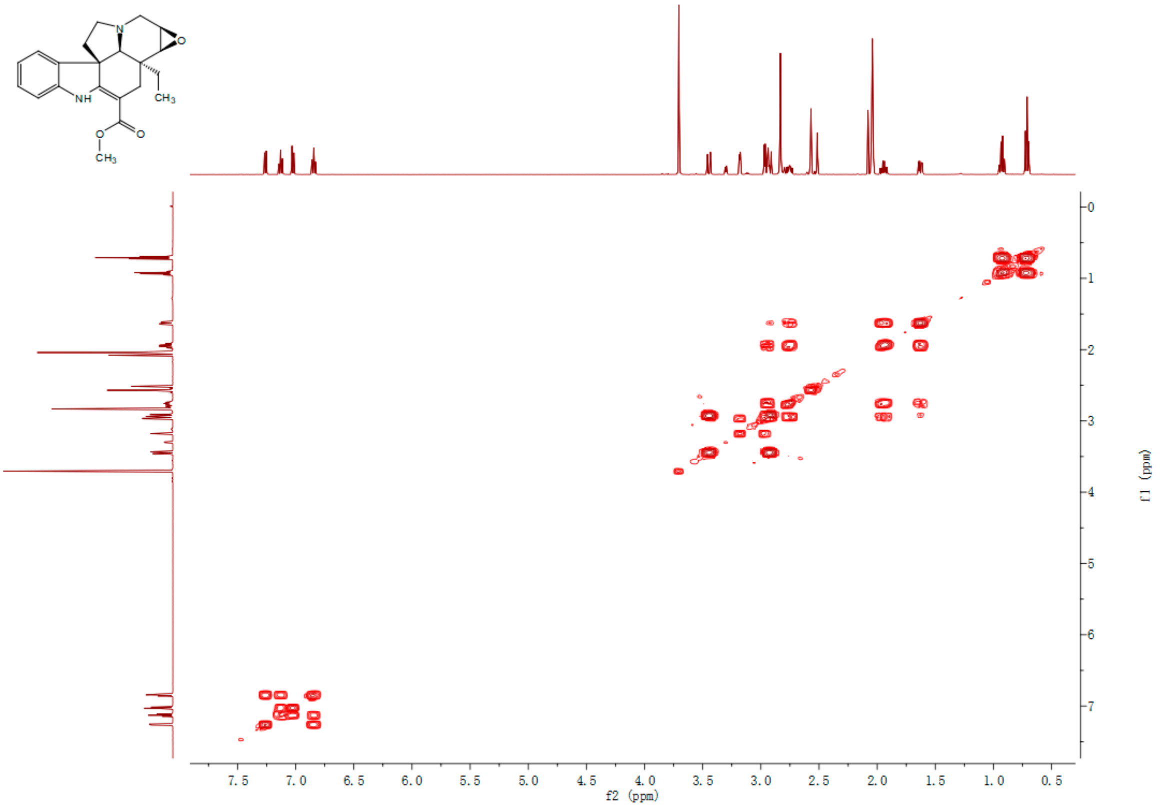
N
O
NH
CH3
O
O
CH3
f2 (ppm)
f1 (ppm)

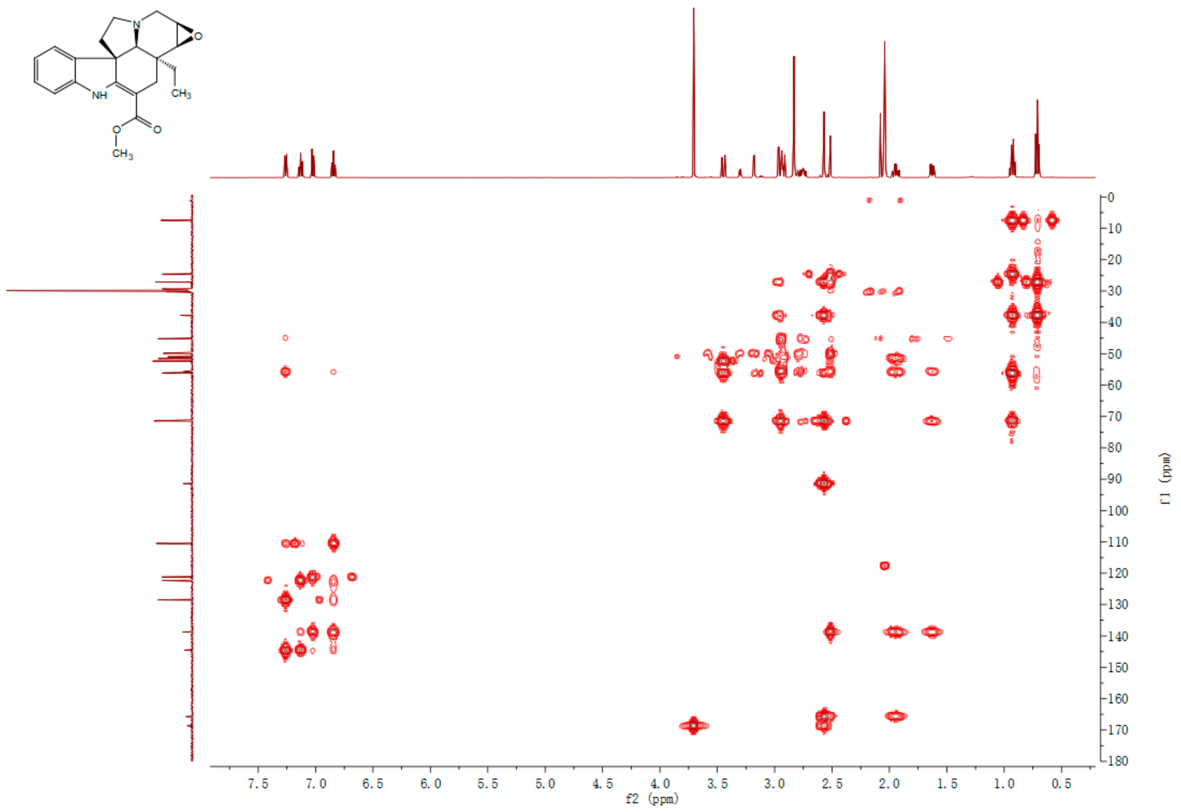

N
O
NH
CH3
O
O
CH3
f2 (ppm)
f1 (ppm)

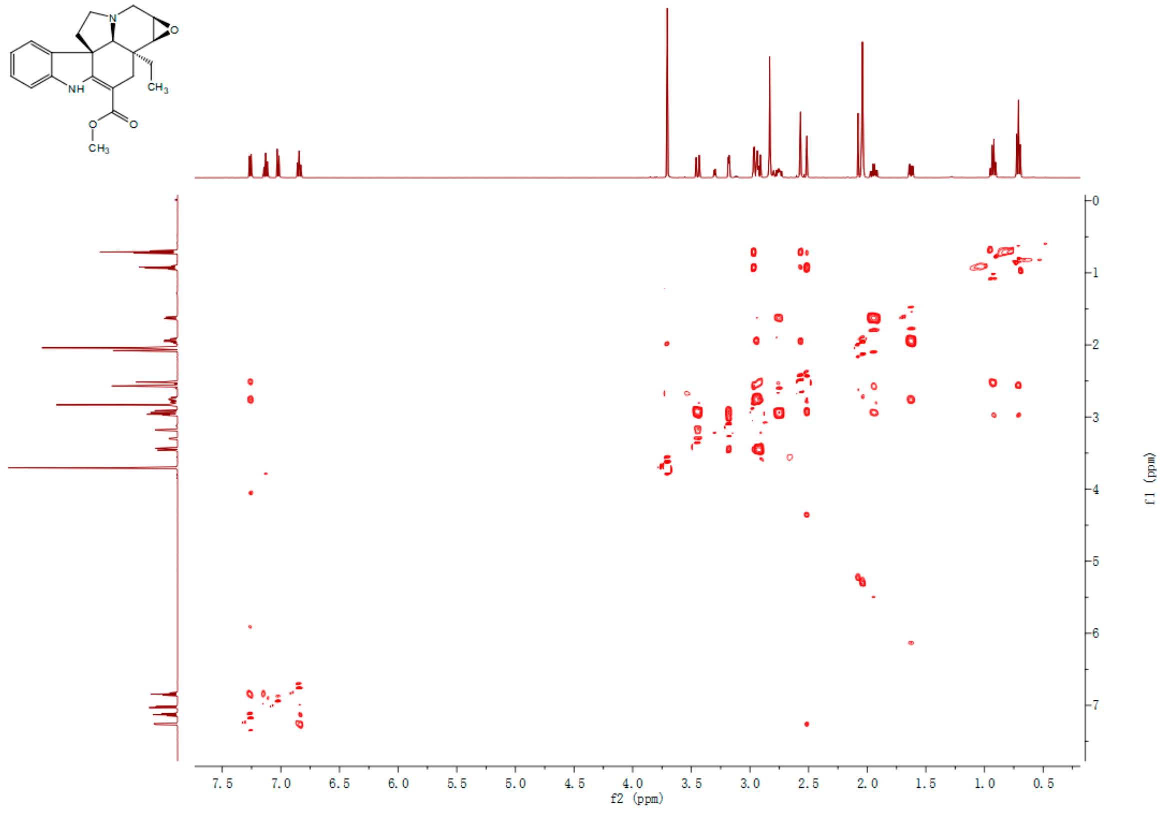

N
O
NH
CH3
O
O
CH3
f2 (ppm)
f1 (ppm)

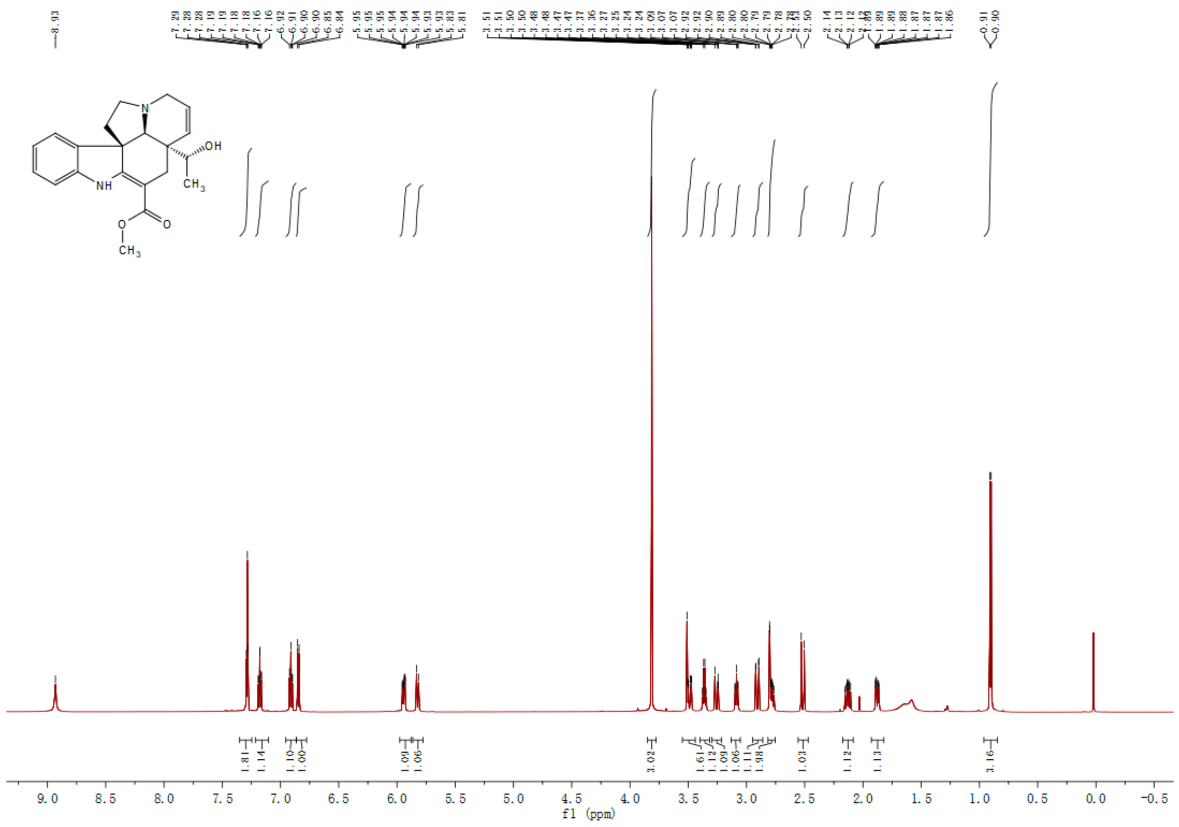

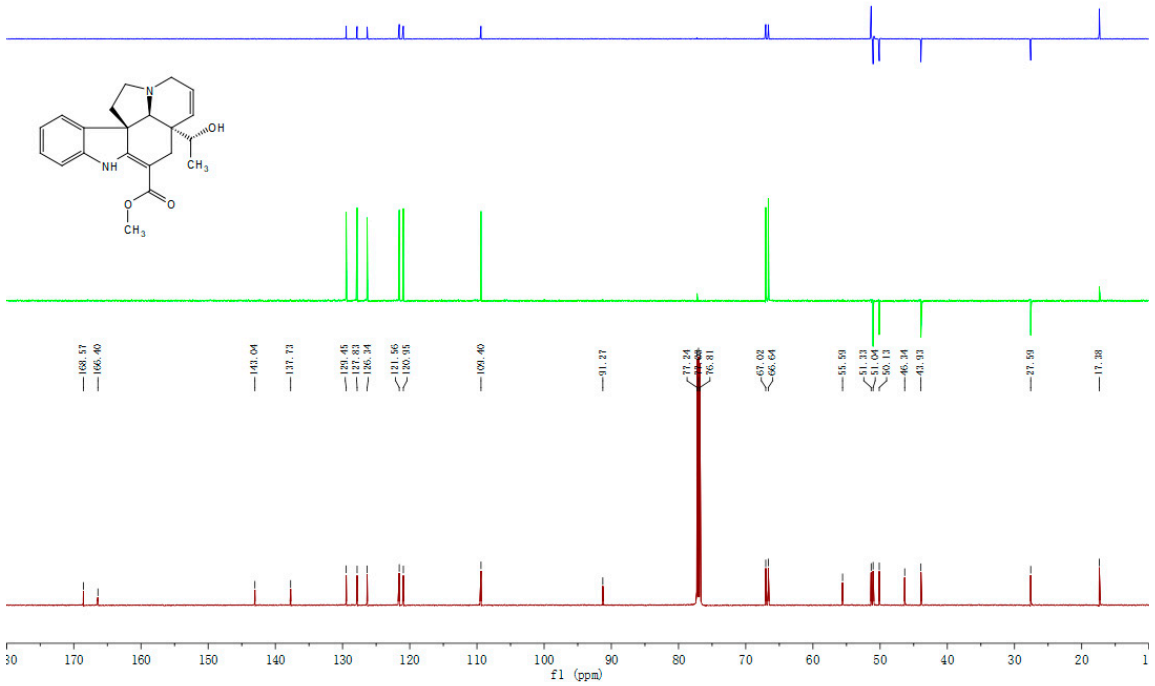

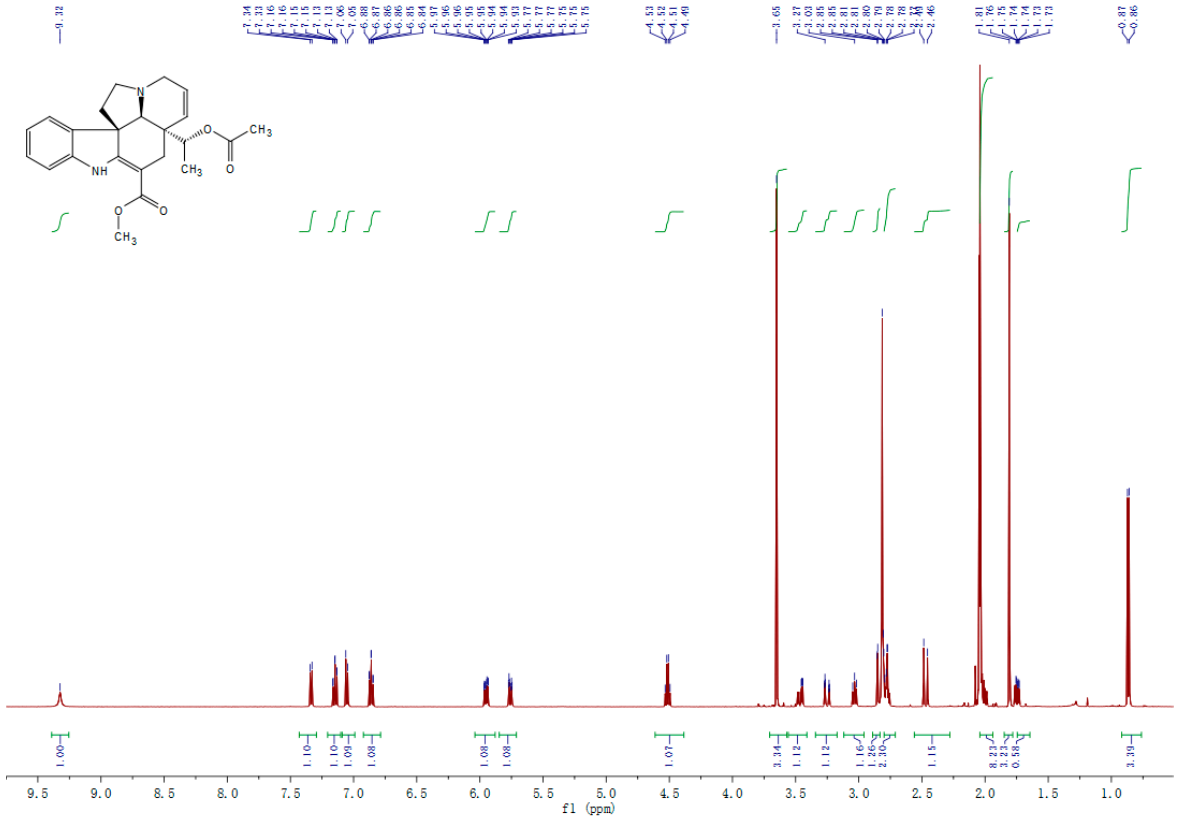

NH
N
O
CH3
CH3
O
O
CH3
fl (ppm)

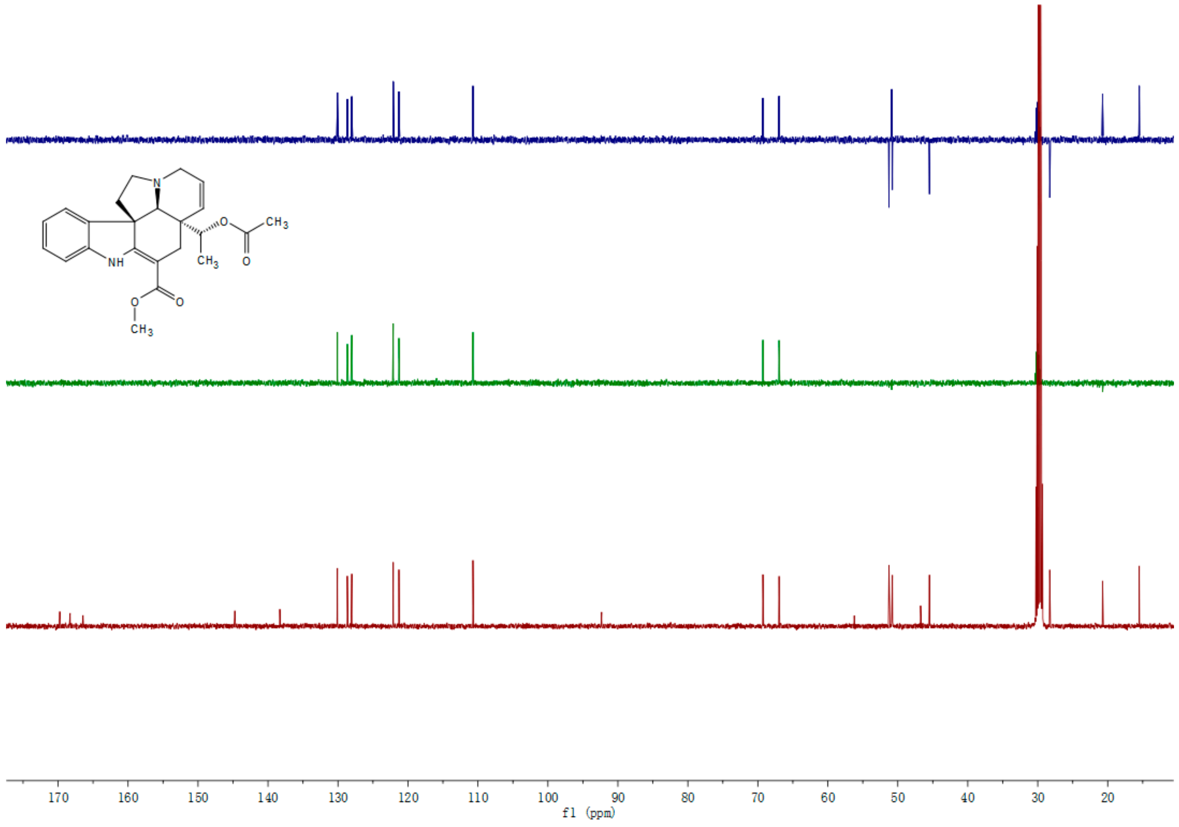

NH
N
O
CH3
CH3
O
O
CH3
fl (ppm)

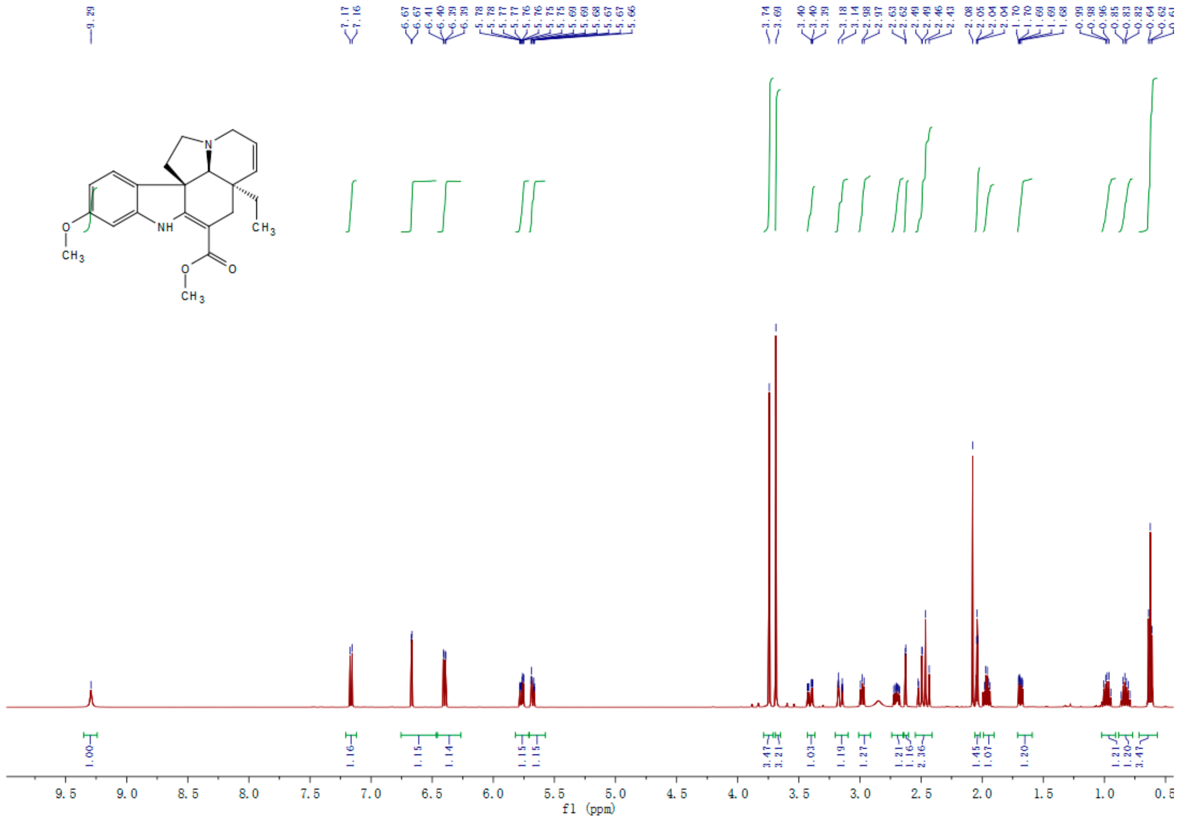
9.29
7.17
7.16
6.67
6.67
6.41
6.40
6.39
6.39
5.78
5.78
5.77
5.77
5.76
5.75
5.75
5.69
5.68
5.68
5.67
5.67
5.66
3.74
3.69
3.40
3.40
3.39
3.18
3.14
2.98
2.97
2.63
2.62
2.49
2.49
2.46
2.43
2.08
2.05
2.04
2.04
1.70
1.70
1.69
1.69
1.68
0.99
0.98
0.96
0.85
0.83
0.82
0.64
0.62
N
O
CH3
NH
CH3
O
O
CH3
1.00
1.16
1.15
1.14
1.15
1.15
3.47
3.21
1.03
1.19
1.27
1.21
1.16
2.36
1.45
1.07
1.20
1.21
1.20
3.47
9.5
9.0
8.5
8.0
7.5
7.0
6.5
6.0
5.5
5.0
4.5
4.0
3.5
3.0
2.5
2.0
1.5
1.0
f1 (ppm)

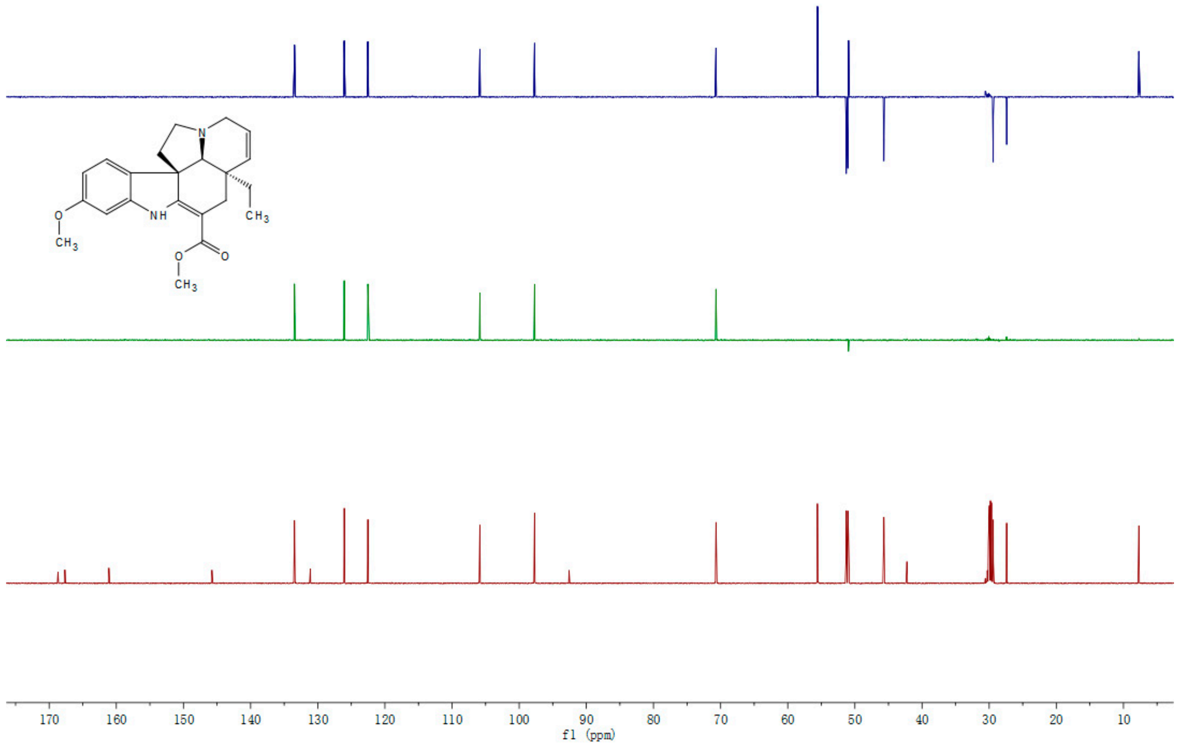
N
O
CH3
NH
CH3
O
O
CH3
170
160
150
140
130
120
110
100
90
80
70
60
50
40
30
20
10
f1 (ppm)

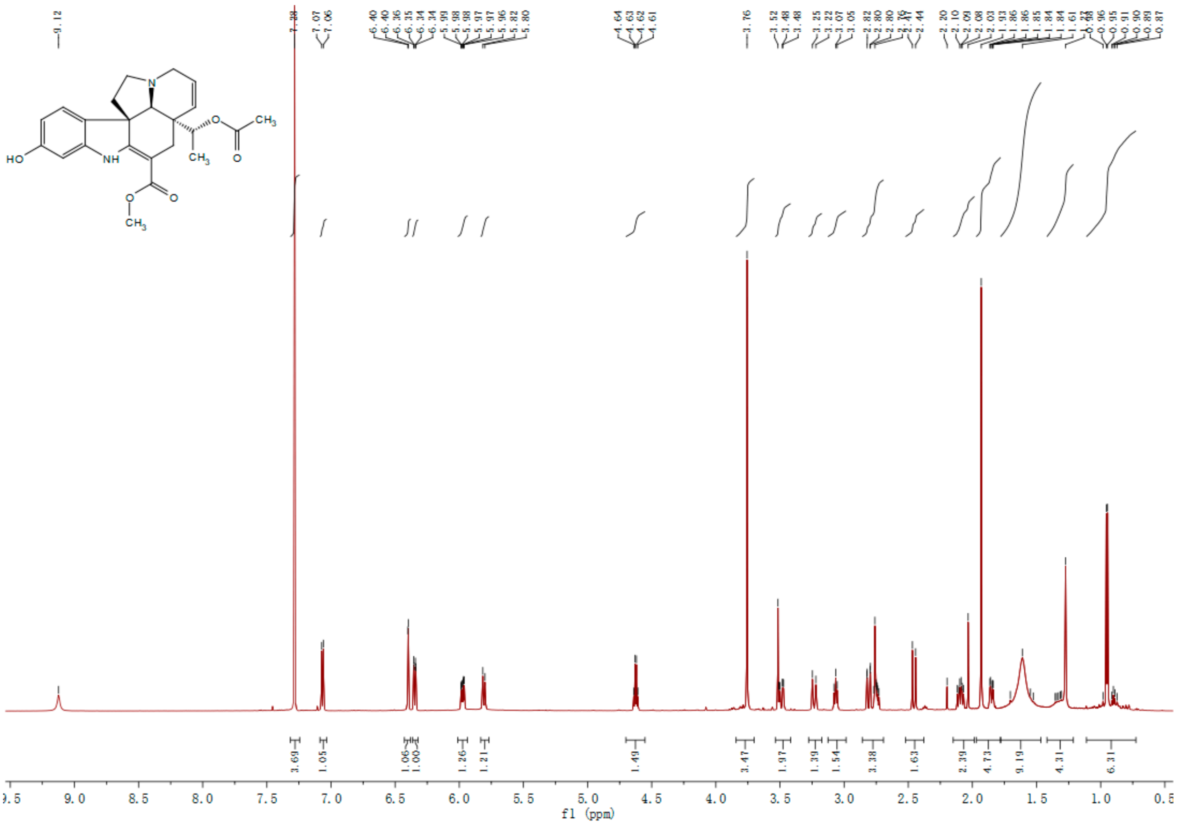
HO
NH
N
CH3
O
f1 (ppm)

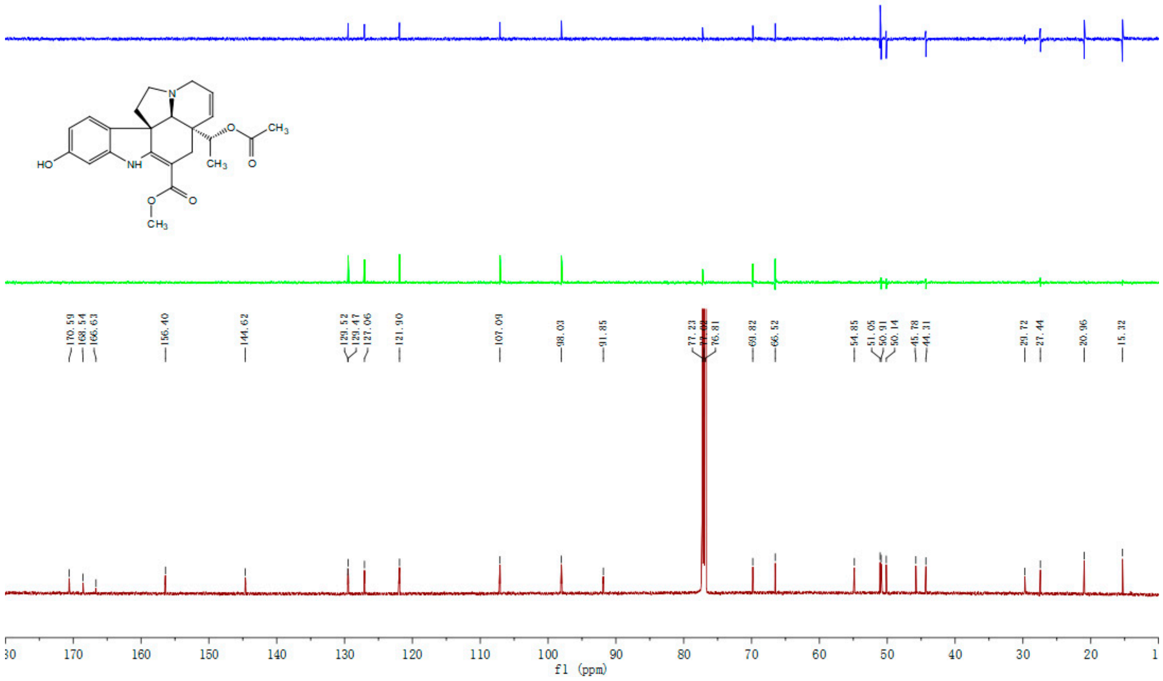
HO
NH
N
CH3
O
170.59
168.54
166.63
156.40
144.62
129.52
129.47
127.06
121.90
107.09
98.03
91.85
77.23
76.81
69.82
66.52
54.85
51.05
50.91
50.14
45.78
44.31
29.72
27.44
20.96
15.32
f1 (ppm)

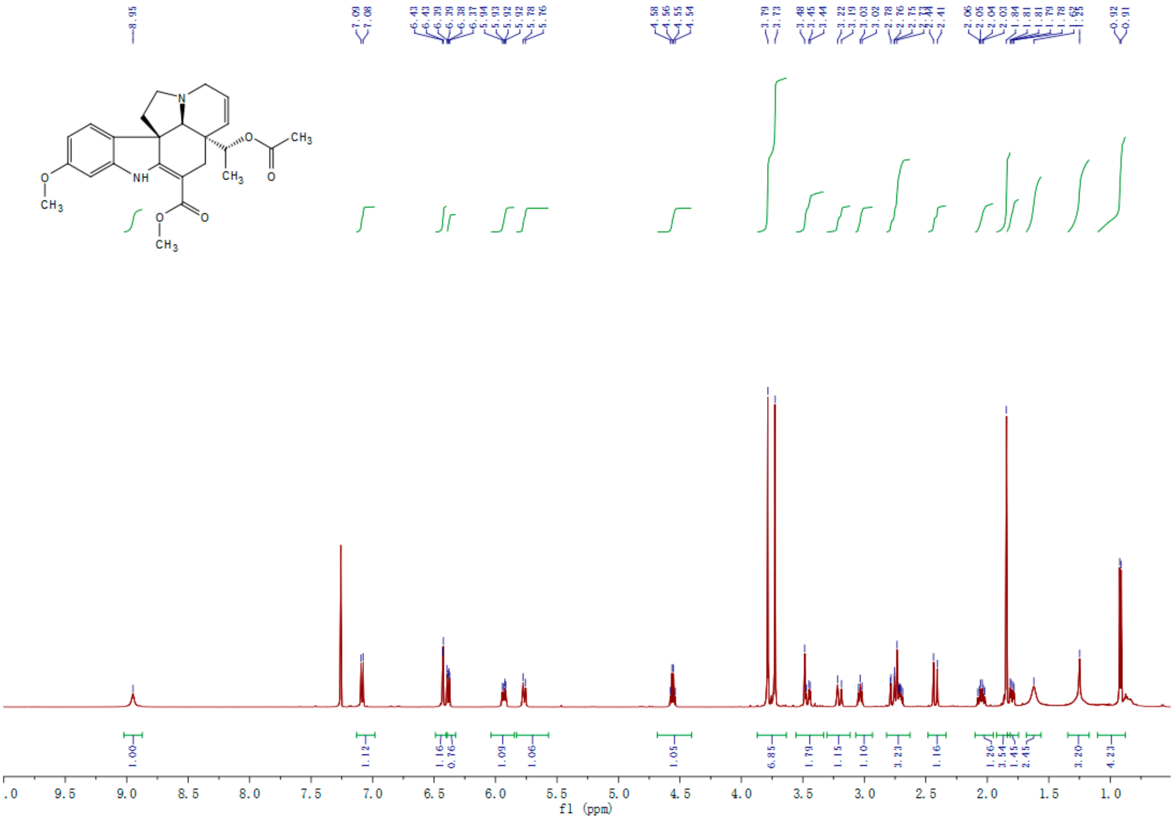

NH
CH3
O
f1 (ppm)

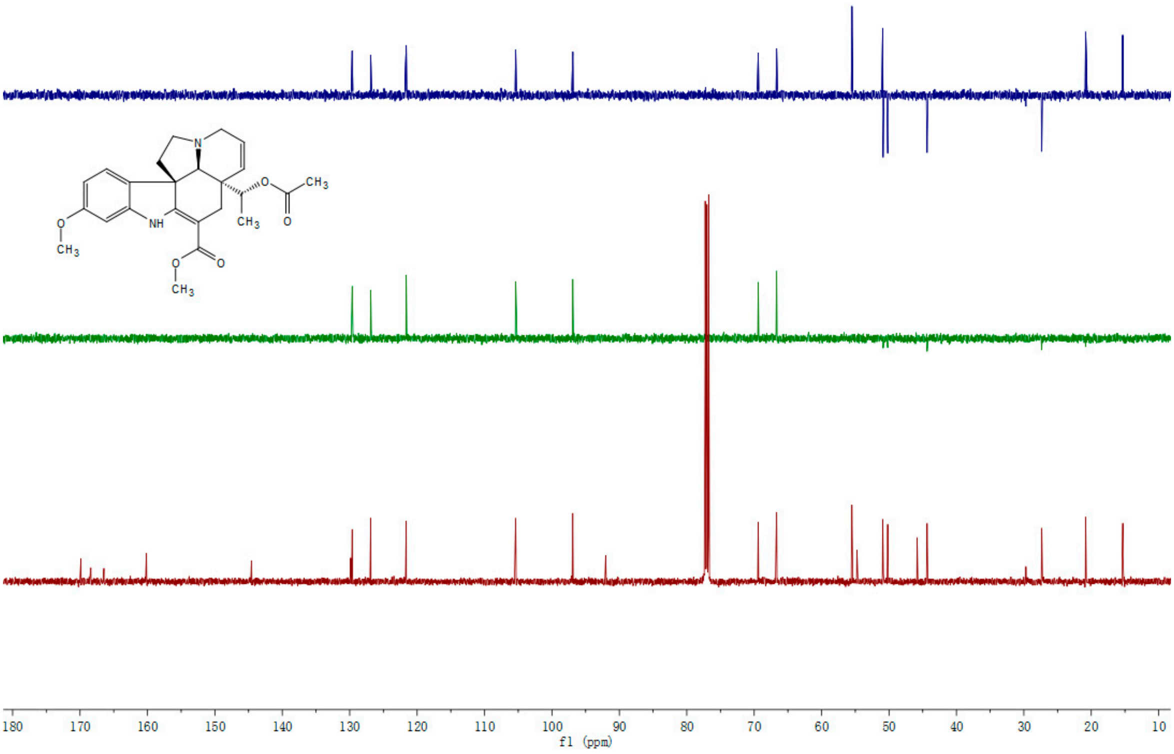

NH
CH3
O
f1 (ppm)
